# Polyaniline Coated Core-Shell Typed Stimuli-Responsive Microspheres and Their Electrorheology

**DOI:** 10.3390/polym10030299

**Published:** 2018-03-10

**Authors:** Yu Zhen Dong, Wen Jiao Han, Hyoung Jin Choi

**Affiliations:** Department of Polymer Science and Engineering, Inha University, Incheon 22212, Korea; 22152270@inha.edu (Y.Z.D.); 22151728@inha.edu (W.J.H.)

**Keywords:** polyaniline, core-shell, microsphere, electrorheological, smart fluid, suspension

## Abstract

Functional core-shell-structured particles have attracted considerable attention recently. This paper reviews the synthetic methods and morphologies of various electro-stimuli responsive polyaniline (PANI)-coated core-shell-type microspheres, including PANI-coated Fe_3_O_4_, SiO_2_, Fe_2_O_3_, TiO_2_, poly(methyl methacrylate), poly(glycidyl methacrylate), and polystyrene along with their electrorheological (ER) characteristics when prepared by dispersing these particles in an insulating medium. In addition to the various rheological characteristics and their analysis, such as shear stress and yield stress of their ER fluids, this paper summarizes some of the mechanisms proposed for ER fluids to further understand the responses of ER fluids to an externally applied electric field.

## 1. Introduction

Electrorheological (ER) fluids are a type of intelligent and smart material, generally consisting of electrically polarizable or semi-conducting materials dispersed in an insulating medium, which are in a fluid-like state in the absence of an electric field and exhibit an almost instantaneous transition to a solid-like state under an applied external electric field [1,2]. Under the application of an external electric field, the initially freely and randomly dispersed particles in the ER fluids aggregate and form chain-like structures [3], which undergo deformation and destruction in a shear flow perpendicular to the electric field and recombine continuously in an applied external electric field. Therefore, the behavior of ER fluids in an electric field, such as shear stress, shear viscosity, and dynamic modulus, can be quite different from those in the absence of an electric field. ER fluids usually exhibit Newtonian fluid-like behavior in the absence of an electric field, in which the shear stress increases linearly with increasing shear rate and the shear viscosity shows an almost constant value. On the other hand, in the presence of an electric field, the ER fluids exhibit Bingham fluid-like behavior, in which the shear stress remains stable in the low shear rate region and increases with increasing shear rate in the high shear rate region. Moreover, the shear viscosity always exhibits obvious shear thinning behavior [4]. Owing to these characteristics, ER fluids have potential applications as dampers [5,6], clutches [7], brakes [8], robotics [9], and finishing [10].

Recently, conducting polymer materials have been studied widely in many fields because of their low density, good thermal and chemical stability, reproducibility, and controllable conductivity [11,12,13]. Polyaniline (PANI), which is one of the most promising and extensively investigated conducting polymers, has been applied extensively to several areas, such as supercapacitors [14], catalysts [15], sensors [16], and biological fields [17], because of its electro-activity, environmental and chemical stability, controllable conductivity, biological compatibility, ease of preparation, and low cost [18]. Furthermore, the properties of ER fluids based on different forms of PANI synthesized by different methods have been studied extensively [19,20,21,22,23,24,25]. On the other hand, pristine PANI also has some defects as an ER material, such as high current density, easily leading to electric breakdown in high electric field strengths; irregular morphology; and colloidal instability. Several methods have been used to improve these problems, such as synthesis of composites [26,27,28,29], and synthesis of PANI with a regular morphology using a template [30,31].

On the other hand, many theoretical models on the ER mechanisms are based on the assumption that the particles dispersed in ER fluids are monodisperse and spherical. Therefore core-shell structured microspheres with a monodispersed spherical core and semi-conductive shell could attract interests for their theoretical approach and as better ER materials.

This paper reviews various PANI-coated core-shell-type microspheres applied to electro-stimuli responsive ER fluids, including their synthetic methods, morphologies, and ER characteristics. In addition, some possible mechanisms proposed for ER behaviors are summarized briefly to further understand ER fluids.

## 2. Core-Shell Typed Microspheres

Core-shell type microspheres are ordered assemblies of materials that are formed by coating other materials with either a chemical bond or other attractive forces between the core and coating shell materials, while possessing a regular spherical morphology. Owing to their unique structural characteristics and the advantages of integrating the properties of both the internal core and external shell materials, core-shell typed microspheres have become an important research direction in recent years and are considered to have a wide range of application prospects in many fields, such as catalysis [32,33], sensors [34], monitors [35], photocatalysis [36], sewage treatment [37], and drug adsorption [38], drug delivery system [39].

Furthermore, the spherical morphology of ER materials is a critical factor in their theoretical models, but many actual ER materials have an irregular morphology. Therefore, to understand the ER mechanism better, many studies have been interested in synthesizing spherical ER materials using various methods. One of them is the synthesis of core-shell typed microspheres by encapsulating irregular materials with a microspherical shape or coating electro-responsive materials on monodispersed spherical core. In addition, a core-shell structured ER material composed of a conductive material and an insulating material can effectively prevent the electrical breakdown that occurs easily during the ER test process. Many ideal core-shell-type microspheres have been applied to ER fluids. For example, Park et al. [40] encapsulated PANI by poly(methyl methacrylate) (PMMA) via a simple physical adsorption route in aqueous media, in which the high conductivity of PANI was controlled by the insulating PMMA shell. Poly(3,4-ethylenedioxythiophene)/poly(styrenesulfonic acid) coated polystyrene (PS) particles via a simple physical adsorption procedure [41], monodisperse polypyrrole (PPy)-coated silica particles using 3-(trimethoxysilyl)propyl methacrylate as a modifying agent to enhance the chemical affinity between core and shell [42], and core-shell structured PS/poly(diphenylamine) via a controlled-releasing process with chemical oxidative polymerization [43] were reported. Park et al. [44] synthesized PPy-coated Fe_3_O_4_ by in situ polymerization, and not only applied these particles to ER fluid, but also to magnetorheological (MR) fluids. In general, ER fluids based on core-shell-type microspheres have attracted considerable interest and deserve further study.

## 3. Electrorheology

### 3.1. Electrorheological Phenomenon

ER fluids are actively controllable smart materials available for many applications. In practice, however, the development of their commercial devices has been hampered by the lack of effective ER fluids except for giant ER fluids with a sufficient yield stress. While an understanding of the mechanism for controlling the ER activity could be one of the keys to developing suitable ER fluids and devices, a range of mechanism or models have been proposed to explain the ER responses.

Initially, Winslow [45] reported that the bridging of fibrillated chains occurs between two electrodes under an electric field, resulting in a significant increase in the viscosity of the suspension. In this fibrillation model, particles are considered to be polarized and be arranged as a dipole aligned with an applied electric field. The neighboring polarized particulates are attracted to each other along the direction of the electric field and are mutually exclusive in the direction perpendicular to the electric field, resulting in an ER effect.

In the case of wet-based moist ER fluids, the electric double layer model and water/surfactant bridge mechanism were proposed to explain the key role of the water content in ER particles on the ER response. Klass et al. [46] proposed that in the presence of water in the ER suspension, the electric double layers wrapped around the particles could be polarized and distorted under an electric field. The distorted electric double layers overlap each other, causing increased electrostatic repulsion, leading to the ER response. On the other hand, Stangroom [47] attributed the ER effect to the formation of an adhesive water bridge among particles. When an electric field is applied, ions from water move out of the pores and migrate to another particle, thus forming a bridge between two particles. Based on this model, Kim et al. [48] proposed a surfactant bridge model in that the surfactant enhances the ER response by increasing the surface polarization at low surfactant concentrations, and a nonlinear ER response due to the formation of surfactant bridges among particles induced by an electric field. Obviously, these mechanisms are limited by anhydrous ER fluids.

**Scheme 1:**
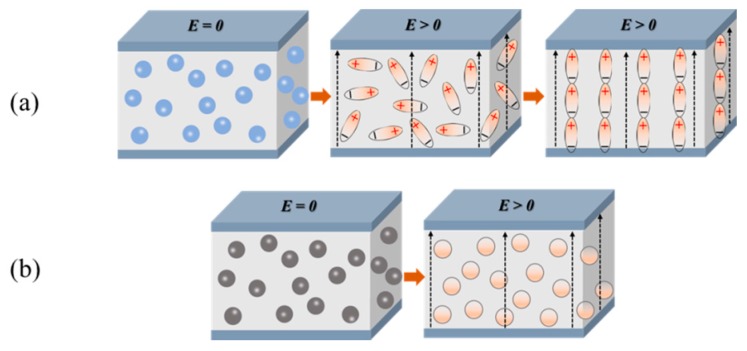
Schematic diagram of the ER particle (**a**) and non-ER particle (**b**) behaviors before and after an external electric field is applied.

For anhydrous dry-based ER fluids, the electrostatic polarization mechanism and conduction model can be used to explain most experimental observations [49]. In the electrostatic polarization mechanism, the ER fluid is believed to consist of a dispersed and continuous phase with different dielectric properties. Based on an idealized ER fluid system, the electrostatic force (*F*) was found to be dependent on the dielectric constant mismatch between the particles and medium as follows:

(1) $$F=k\varepsilon_{m}{\left(2r\right)}^{2}f^{2}E^{2}S$$

where *k* is a constant; *r* is the particle radius; *S* is a factor related to the particle microstructure; f=(g−1)/(g+2); and $g=\varepsilon_{p}/\varepsilon_{m}$, in which $\varepsilon_{m}$ is the dielectric constant of the continuous phase and $\varepsilon_{p}$ is the permittivity of a hard dielectric sphere. Many experimental observations confirmed that the particles tend to form chains that span the electrode gap and the yield stresses vary with the square of the applied electric field strength [50]. In addition, Hao et al. [51,52] proposed an extended electrostatic polarization model-dielectric loss model. The ER response is considered to be divided into two dynamic processes. The first step is the dielectric constant-dominated particle polarization process and the second step is particle turning that is determined by the particle dielectric loss. Under an applied electric field, both ER particles and non-ER particles could be polarized but only ER particles can form a fibrillated bridge between two electrodes, as shown in Scheme 1. Nevertheless, these mechanisms are limited by dc or low frequency ac electric fields, in which case, the conductivity mismatch between the particle and liquid phase is considered to be a dominant factor rather than the dielectric constant mismatch [53]. Thereby, Atten [54] and Foulc [55] proposed a conduction model to explain the case when both the particle and liquid phase are conductive. This model could predict the current density, yield stress, and temperature dependence of the ER fluids. On the other hand, because the change in the microstructure after the electric field was applied was not considered, this model was applicable only when used in static situations where the particle microstructure had been fully formed [56].

### 3.2. Electrorheological Particles

A variety of particulates, ranging from inorganic to organics to polymers, have been proposed for the use as ER particles. Many chemical and physical methods, including sol-gel, Pickering emulsion, hydrothermal, etc., are used for their synthetic process. Although the particulate materials should be selected based on their physical and chemical properties, ER particles generally possess a relatively high dielectric constant and a low conductivity (<10^−6^ S/m) with their densities almost matching that of the corresponding dispersing phases.

Under an applied electric field, the particle size and shape affect the ER performance. Particles with a number average size of 0.1 to 100 μm are commonly used in ER fluids. Particles that are too small or too large are expected to exhibit weak ER performance, as Brownian motion or sedimentation would have a dominantly effect in the formation of fibrillation bridges. The size of the particles also depends on the gap between the electrode elements in the ER device to be used. Nevertheless, many experimental results indicate that there is no simple dependency between the particle size and ER performance. Cho et al. [57] prepared three different core sizes of monodisperse polymeric microspheres, and examined the particle size effect on the ER performance. The results showed that the ER performance increased with increasing core particle size, and a larger particle size ranging from 1 to 10 µm is advantageous for the ER property if their electrical properties are similar. In addition, the computer simulation result showed that the field-induced shear stress could be proportional to the cube of the particle diameter [58]. On the other hand, a large enhancement of yield stress was achieved by decreasing the size of barium titanyl oxalate nanoparticles coated with urea for giant ER fluids [59].

The particles used in an ER fluid can be in the form of spherical, fibrous, rod, sheet, and core-shell structures. The dielectric property of a suspension is closely related to the shape of dispersed particles [60]. The interfacial polarizability of particles may increase with increasing dielectric constant, resulting in a stronger ER response [61]. Liu et al. [62] synthesized core-shell structured snowman-like particles and suggested that this snowman-like particle-based ER fluid exhibited a larger yield stress and shorter relaxation time than spherical particles owing to its polarization along the long axis in a short time. The dynamic modulus increased almost linearly with increasing aspect ratio of the particle [63]. The fibrous or ellipsoidal particles may be advantageous to enhancing the ER performance.

As a new discovery, the concept of a giant ER fluid, which can be distinguished from conventional dielectric ER fluids, has been proposed. Because most practical applications require a strong yield or shear behaviors of ER fluids, the relatively low yield behavior of conventional ER fluids limits their applications to a great extent. Wen et al. [64] developed urea-coated barium titanyl-oxalate nanoparticle [NH_2_CONH_2_@BaTiO(C_2_O_4_)_2_]-based ER fluid with a yield stress of up to 130 kPa, which far exceeded the theoretical limits predicted by conventional dielectric ER fluids. This giant ER fluid displays a near-linear dependence of the static yield stress on the applied electric field, as well as an opposite dependence on the particle size. Therefore, the rheological properties of giant ER fluids cannot be explained fully by conventional ER models. To understand the giant ER effect, Yin et al. [65] synthesized a new ER material of mesoporous cerium-doped TiO_2_ with a high surface area and robust crystalline pore walls, and observed its giant ER activity with a high yield stress of 70 kPa. Lu et al. [66] synthesized a Ca-Ti-O-based ER liquid by a wet chemical method, and proposed a polar molecule-type ER (PM-ER) fluid. They developed a polar molecular orientation and bonding model that suggested there are two types of interactions among particles: dipole-dipole interactions and dipole-charge interactions. Recently, Hong and Wen [67] adopted six different oil samples to investigate the wetting characteristics of giant ER fluids. They suggested that the carrier liquid played a key role in the giant ER effect, in which the highest yield stress of the giant ER fluid was obtained when hydrogenated silicone oil was used as the continuous phase. Certainly, great advancement has been made in understanding the mechanisms of typical fluids and giant ER fluids using the current theoretical models. Future studies will still require the establishment of different models for theoretical and experimental research.

## 4. Polyaniline-Coated Core-Shell Structured Microspheres

### 4.1. Inorganic Core

#### 4.1.1. Silica (SiO_2_)

Silica (SiO_2_) particles are used widely in the fields of drug delivery [68,69], food industry [70,71], catalytic [72], sensor [73], biochemical [74], etc. [75,76] because of their special porous structure, biocompatibility, colloidal stability, low cost, and easy preparation. In addition, owing to their excellent reinforcing properties and regular spherical morphology, SiO_2_ particles are considered very promising partners for the preparation of polymer-coated composite materials. The preparation of conductive polymer-coated SiO_2_ composites has attracted considerable attention, such as PANI [77,78], polythiophene [79], polypyrrole [80], poly(2-methylaniline) [81], and poly(3,4-ethylenedioxythiophene) (PEDOT) [82]. Furthermore, such particles have great potential as ER materials.

**Scheme 2:**
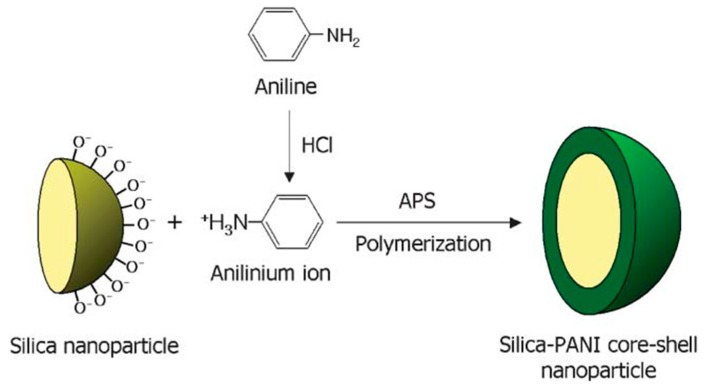
Synthetic procedure of SiO_2_/PANI core-shell nanoparticles. (Jang et al. [84], Copyright The Royal Society of Chemistry, 2006).

Trlica et al. [83] applied core-shell-type SiO_2_/PANI microspheres to ER materials by coating PANI on the surface of porous spherical SiO_2_ with a mean particle size of 15 μm through an in situ chemical oxidative polymerization without a surfactant. The as-synthesized SiO_2_/PANI microspheres were treated with hydrochloric acid (HCl) and ammonium hydroxide (NH_3_·H_2_O) to obtain SiO_2_ coated with a protonated PANI and PANI base, respectively. Jang et al. [84] also synthesized SiO_2_/PANI core-shell spherical nanoparticles with a diameter less than 30 nm by the in situ polymerization of aniline on the SiO_2_ surface, which has a mean diameter of 22 nm. Scheme 2 shows the synthetic procedure, in which the protonated aniline monomer is positively charged and adsorbed onto the negatively charged SiO_2_ surface through electrostatic interactions. After adding the ammonium persulfate (APS) initiator, the aniline monomer begins to polymerize and form a PANI shell on the SiO_2_ core.

Hong et al. [85] dispersed these particles in silicone oil to examine the ER properties and compared these properties with those of other conducting polymer (polythiophene (PT), polypyrrole (PPy), and poly(ethyldioxythiophene) (PEDOT))-coated SiO_2_ core-shell nanoparticle-based ER fluids. The results showed that the SiO_2_/PANI-based ER fluid has the highest yield stress, which will be discussed later.

**Scheme 3:**
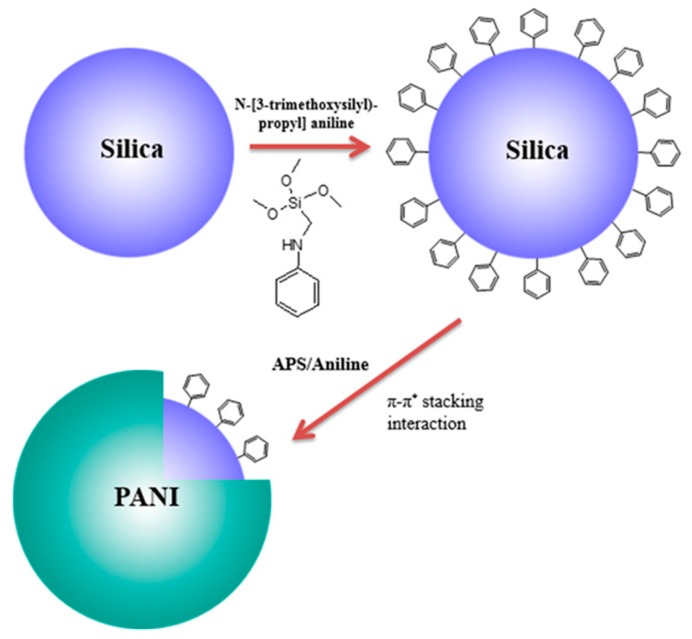
Schematic diagram of the PANI coating on the silica surface. (Park et al. [86], Copyright IOP Publishing Ltd., 2016).

**Figure 1:**
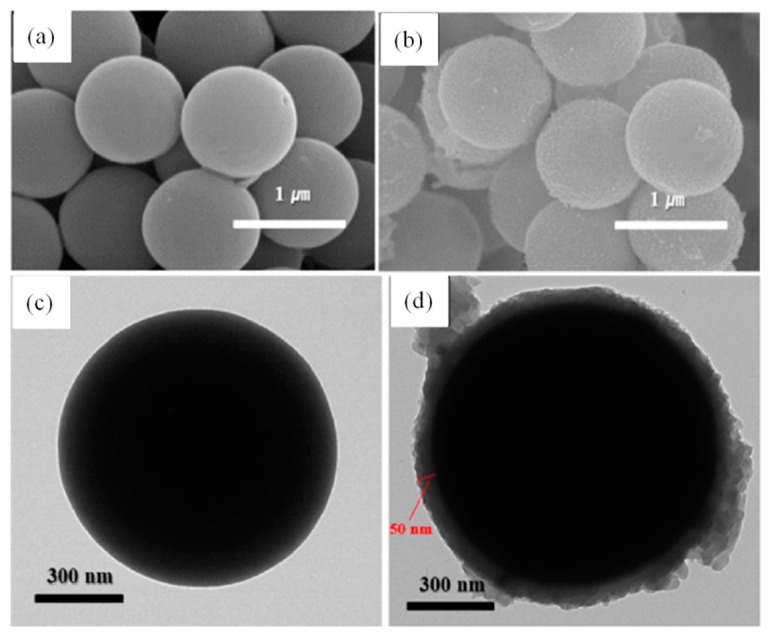
SEM (**a**,**b**) and TEM (**c**,**d**) images of pure SiO_2_, and SiO_2_/PANI core–shell microspheres. (Park et al. [86], Copyright IOP Publishing Ltd., 2016).

On the other hand, Park et al. [86] coated PANI uniformly over a silica surface by modifying the silica in advance with *N*-[(3-trimethoxysilyl)-propyl] aniline. Scheme 3 shows a graphical diagram of the synthetic process of SiO_2_/PANI, in which the aniline monomers are adsorbed on the surface of modified silica particles through π-π* stacking interactions, and polymerization starts after adding APS. Figure 1 shows SEM and TEM images of SiO_2_ and SiO_2_/PANI, after coating PANI, the surface of the particles become rougher and both SiO_2_ and SiO_2_/PANI microspheres showed a diameter of approximately 1 μm, where the thickness of the PANI shell was approximately 50 nm by TEM analysis.

#### 4.1.2. Ferric Oxide (Fe_2_O_3_)

Ferric oxide (Fe_2_O_3_) has been studied extensively in many applications, such as photocatalysis [87,88], wastewater treatment [89], biomedical [90], microwave absorption [91], sensors [92], and lithium-ion battery anodes [93]. A variety of Fe_2_O_3_ structures, such as cube-shaped [94], rod-shaped [95], pine-leaf-shaped [96], and flower-like [97], have been fabricated successfully by different steps. Flower-like Fe_2_O_3_ has attracted considerable attention because of its special structure.

**Figure 2:**
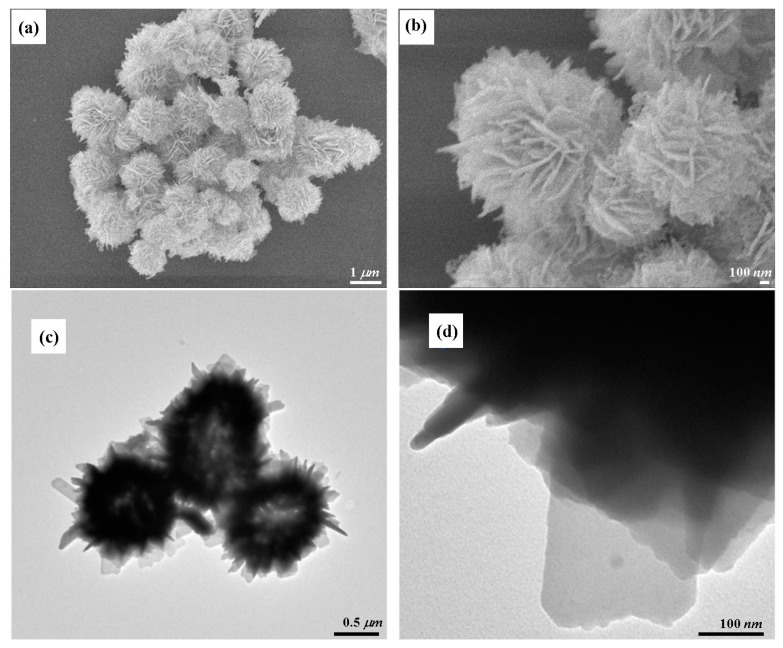
SEM (**a**,**b**) and TEM (**c**,**d**) images of Fe_2_O_3_/PANI nanocomposite particles. (Tian et al. [98], Copyright Elsevier, 2016).

Tian et al. [98] synthesized flower-like Fe_2_O_3_/PANI core/shell nanoparticles consisting of special hierarchical structures that give them special physical and chemical properties using a two-steps method. These novel core/shell structured particles were applied as an ER material. The monodispersed flower-like Fe_2_O_3_ was prepared by a high temperature refluxing method using ferric chloride hexahydrate, urea, tetrabutyl ammonium bromide, and ethylene glycol. The final flower-like Fe_2_O_3_ particles were obtained by calcining the as-obtained flower-like sample at 550 °C for 2 h. The core/shell-structured Fe_2_O_3_/PANI were synthesized by in situ polymerization with cetyltrimethyl ammonium bromide (CTAB) as a surfactant. As shown in Figure 2, the synthesized flower-like Fe_2_O_3_/PANI nanoparticles were uniform and monodispersed. The PANI fill in the sheets of flower-like Fe_2_O_3_ and coat its surface without destroying the spherical particle shape and make the surface more cluttered and rougher than pure Fe_2_O_3_.

#### 4.1.3. Iron Oxide (Fe_3_O_4_)

Iron oxide (Fe_3_O_4_) has attracted widespread interests in many fields, such as lithium-ion batteries [99], electromagnetic shielding [100], adsorbents [101,102], catalysts [103,104], and so on [105,106], because of its paramagnetic, low cost, large surface area, biocompatibility, environment-friendly, and electrochemical stability [107,108].

**Figure 3:**
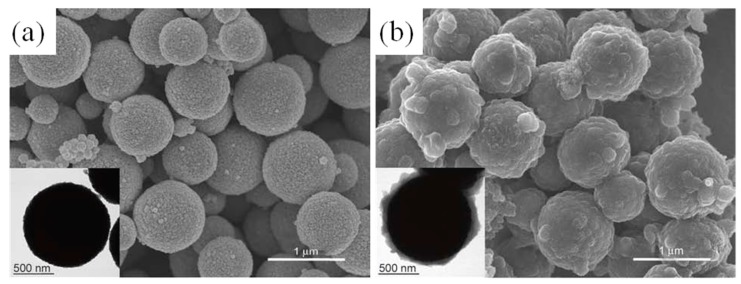
SEM and TEM (inset) images of (**a**) Fe_3_O_4_, (**b**) Fe_3_O_4_@PANI (Sim et al. [109], Copyright BME-PT, 2015).

Sim et al. [109] fabricated PANI-coated iron oxide (Fe_3_O_4_@PANI) spherical particles with acidification and applied them to both ER and MR materials. The spherical Fe_3_O_4_ particles were synthesized by a solvothermal process using ferric chloride hexahydrate, sodium acetate, and ethylene glycol. Fe_3_O_4_ particles were acidified by HCl. After acidification, the aniline monomer was adsorbed onto the surface of the Fe_3_O_4_ core due to electrostatic interactions and hydrogen bonding. PANI can then grow on the Fe_3_O_4_ core through a chemical oxidative polymerization method and form core-shell structured microspheres. Figure 3 presents the morphology of Fe_3_O_4_ (a) and PANI-coated Fe_3_O_4_ (b). Pure Fe_3_O_4_ is spherical with a grainy surface that changes significantly after coating with PANI. The core-shell structure of Fe_3_O_4_@PANI can be confirmed by TEM.

#### 4.1.4. Titanium Oxide (TiO_2_)

Titanium oxide (TiO_2_) particles have many benefits, including of its low price, chemical stability, dielectric properties, and easy synthesis [110], being applied into many areas, such as solar cells [111], photocatalysis [112,113], and environmental restoration [114,115].

Inorganic-organic core-shell-structured TiO_2_/PANI composite can combine the high chemical stability and dielectric properties of TiO_2_ and the tunable electric conductive property and low density of PANI, which is considered a potential ER material. Wang et al. [116] fabricated a core-shell structured TiO_2_/PANI nanocomposite via in situ oxidative polymerization in the presence of a surfactant after the preparation of TiO_2_ nanospheres. The TiO_2_ was prepared by the controlled hydrolysis of tetrabutyl titanate and calcination at 550 °C to obtain single-crystal TiO_2_. The type of surfactant (CTAB), polyvinyl pyrrolidone (PVP), polyvinyl alcohol (PVA), sodium dodecyl sulfonate (SDS), amount of aniline monomer, and type of protonic acid (citric acid, hydrochloric acid (HCl) and acetic acid (HAc)) on the morphology of the TiO_2_/PANI particles were studied; CTAB was found to be the best of the applied surfactants. When using CTAB as a surfactant and HCL as a protonic acid, PANI can coat the TiO_2_ surface well, and produce interesting PANI nanorings.

**Scheme 4:**
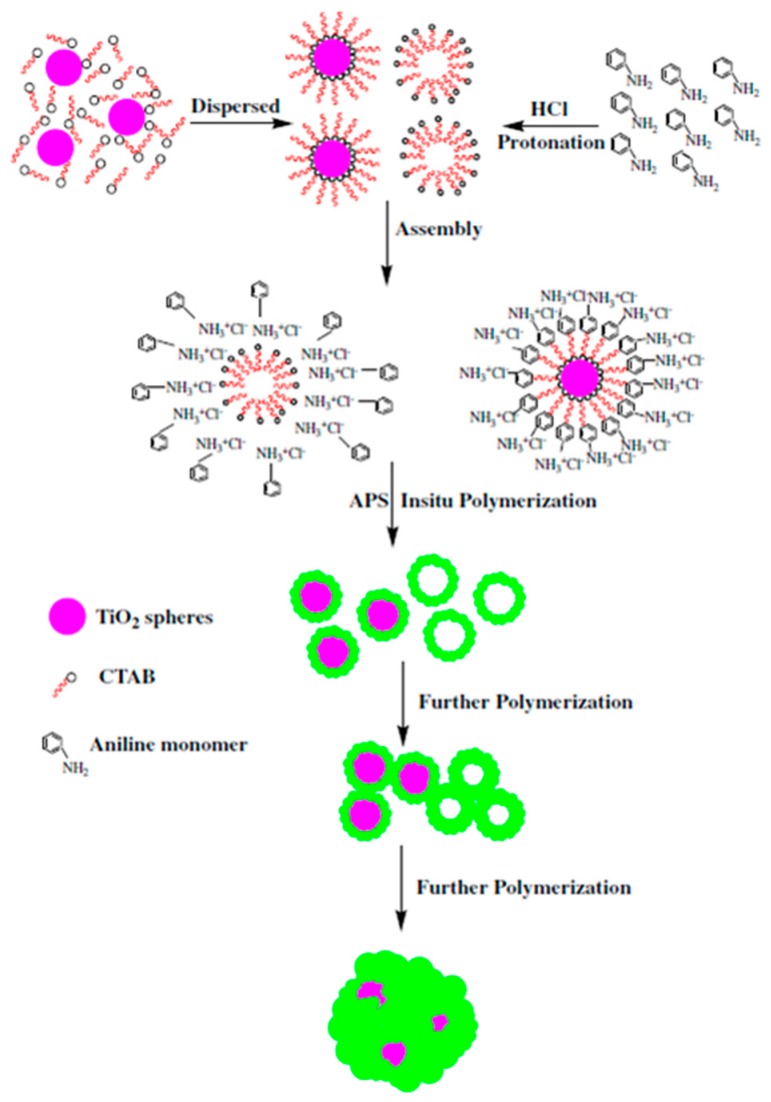
Proposed schematic growth mechanisms for the formation of TiO_2_/PANI nanocomposite. (Wang et al. [116], Copyright Elsevier, 2013).

Scheme 4 shows a probable schematic diagram of the TiO_2_/PANI generation mechanism using CTAB and HCl. While the TiO_2_ nanospheres and CTAB surfactant are dispersed in an aqueous solution, CTAB will be adsorbed to the surface of hydrophilic TiO_2_ nanospheres or form spherical micelles without TiO_2_. With the addition of a protonated aniline monomer, two types of CTAB spherical micelles with and without TiO_2_ will induce the assembly of a protonated aniline monomer on CTAB. The in situ polymerization of aniline will then occur after the addition of an initiator, APS, to form the core-shell structured TiO_2_/PANI nanocomposites or hollow PANI nanorings. If the dose of aniline monomer increases, further polymerization will cause the growth of ring-like PANI, which will reduce the pore size of the PANI nanorings and cause the rings to interconnect and form nanofiber webs. With the continued increase in the amount of monomer, excessive polymerization leads to the aggregation of PANI and the formation of thicker PANI shells.

Tian et al. [117] synthesized an anisotropic peanut-like TiO_2_/PANI core/shell nanocomposite via a three-step method. First, monodispersed amorphous TiO_2_ nanospheres were prepared by the controlled hydrolysis of tetrabutyl titanate, similar to that reported by Wang et al. [116]; the reaction equation is shown below:

Ti(OC_4_H_9_)_4_ + 4H_2_O = Ti(OH)_4_ + 4C_4_H_9_OH (2)

**Figure 4:**
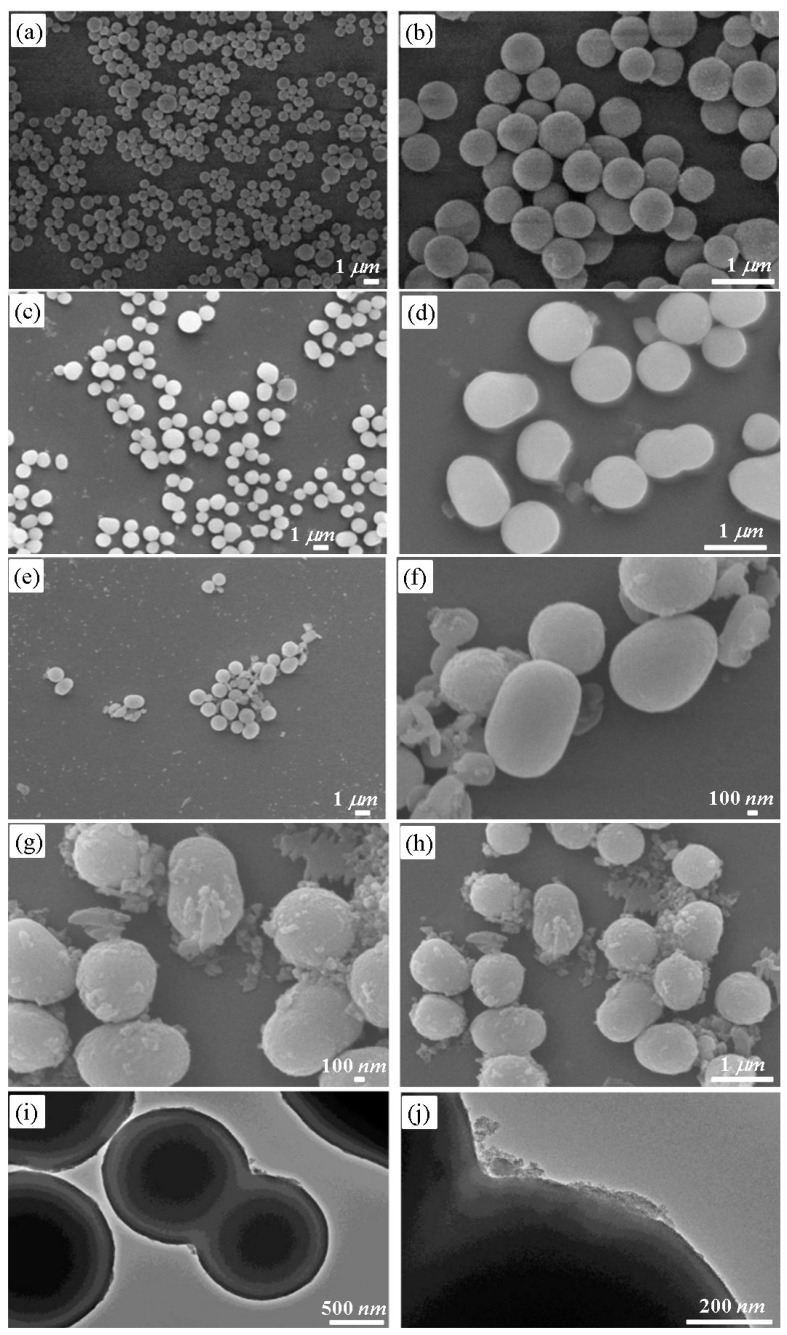
SEM images of (**a**,**b**) amorphous TiO_2_ under different magnifications; (**c**,**d**) anisotropic peanut-like TiO_2_ obtained using glacial acetic acid, (**e**,**f**) oleic acid. SEM (**g**,**h**) and TEM (**i**,**j**) images of anisotropic peanut-like titanium TiO_2_/PANI core/shell nanocomposite. (Tian et al. [117], Copyright Elsevier, 2016).

On the other hand, as-obtained monodispersed TiO_2_ nanospheres are amorphous and have hydroxyl groups (–OH) on their surface, while anisotropic peanut-like TiO_2_ nanospheres were obtained using a weak acid to etch the monodispersed TiO_2_ spheres. Tian et al. [117] used glacial acetic acid (GAA) and oleic acid. Figure 4a,b show SEM images of amorphous TiO_2_ and Figure 4c–f show SEM images of anisotropic peanut-like TiO_2_ obtained using glacial acetic acid and oleic acid, respectively. The results show that GAA has a better etching effect, and can etch the amorphous TiO_2_ nanospheres into more regular peanut-like TiO_2_ nanospheres.

Finally, PANI was coated on the surface of the anisotropic peanut-like TiO_2_ using an in situ polymerization method with CTAB as a surfactant. Figure 4g–j show SEM and TEM images of anisotropic peanut-like titanium TiO_2_/PANI core/shell nanocomposite, respectively. A thin polyaniline layer was coated on the surface of peanut-like TiO_2_.

### 4.2. Polymeric Core

#### 4.2.1. Poly(methyl methacrylate)

Poly(methyl methacrylate) (PMMA), which is usually synthesized by a dispersion polymerization method, has potential use as a core in core-shell structure because of its regular spherical morphology and mono-dispersity [118,119]. Therefore, PANI-coated mono-dispersed spherical PMMA core has been applied widely to ER materials.

Cho et al. [120] prepared monodispersed PMMA spheres with a diameter of approximately 2 μm by the dispersion polymerization of methyl methacrylate (MMA) monomers in a methanol medium with 2,2-azobisisobutyronitrile (AIBN) as an initiator and PVP as a stabilizer, and then coated with PANI by the chemical oxidative polymerization of aniline monomers on the surface of PMMA using sodium dodecyl sulfate (SDS) as a surfactant. They examined the ER properties of this PANI-coated PMMA (PMMA/PANI) particle-based ER fluid. Based on this approach, Cho et al. [57] continued to study the effect of the particle size on the ER characteristics of PMMA/PANI-based ER fluid. They synthesized PANI-coated PMMA particles with a diameter of 2, 4.5 and 9 μm, respectively, and compared the ER characteristics of these particle-based ER fluids.

**Scheme 5:**
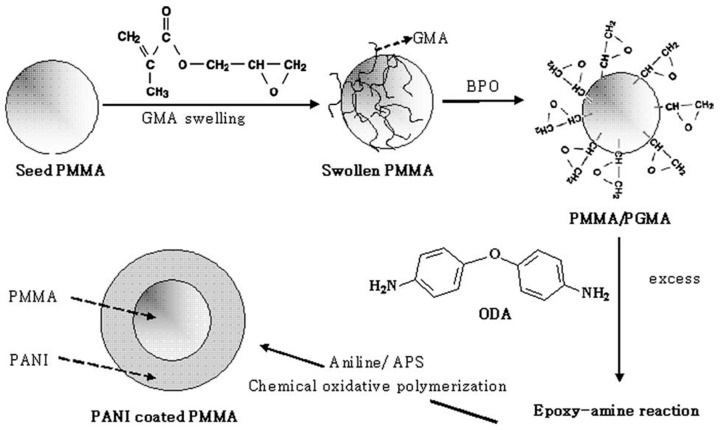
Schematic diagram of the polymerization procedure of PMMA/PANI. (Lee et al. [121], Copyright Elsevier, 2005).

Furthermore, Lee et al. [121] synthesized PMMA/PANI microspheres with the graft polymerization of aniline, improving the adhesion between PANI and PMMA and forming a uniform PANI shell thickness. Scheme 5 presents a schematic diagram of the synthetic procedure. The seed PMMA particles with a diameter of 6.5 μm were prepared using a dispersion polymerization method and swollen by glycidyl methacrylate (GMA) and benzoyl peroxide (BPO) initiator. When the temperature was increased to 75 °C, GMA began to polymerize on the PMMA surface to form poly(glycidyl methacrylate) (PGMA), which contains glycidyl groups. The oxydianiline (ODA) and glycidyl groupsthen undergo an epoxy-amine reaction to graft the aniline functionality onto PMMA cores. Finally, the aniline polymerizes on the surface of PMMA with APS as an initiator and PVA as a stabilizer to form a PANI shell.

**Figure 5:**
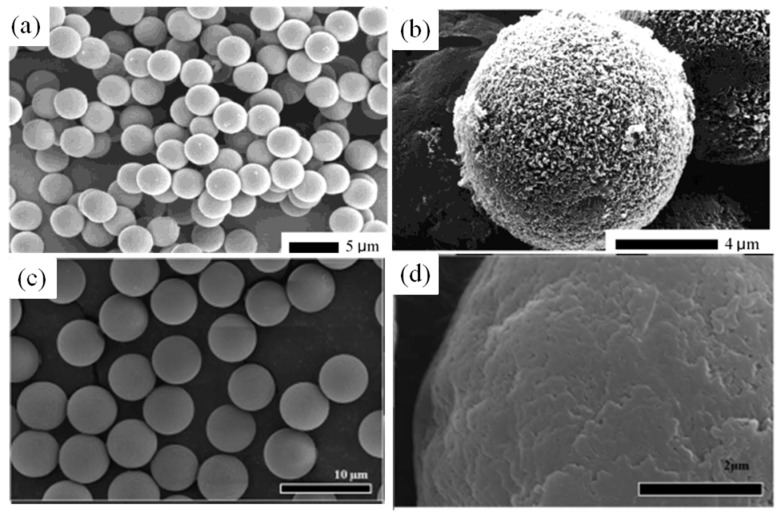
SEM images of (**a**) PMMA and (**b**) PMMA/PANI particles synthesized by Cho M.S. et al. [57] (Copyright American Chemical Society, 2003) and (**c**) PMMA and (**d**) PMMA/PANI particles synthesized by Fang et al. [122] (Copyright The Royal Society of Chemistry, 2011), respectively.

Fang et al. [122] added the ethylene glycol dimethacrylate (EGDMA) as a crosslinking agent on PMMA core to enhance further the mechanical strength of the PMMA/PANI particles; the other synthetic steps are the same as above (Lee et al. [121]). Figure 5 shows SEM images of PMMA and PMMA/PANI particles synthesized by Cho et al. [57] and Fang et al. [122]. The PMMA particles were spherical and monodispersed with a smooth surface, and after coating with PANI, the surface of particles became rougher.

#### 4.2.2. Poly(glycidyl methacrylate)

**Scheme 6:**
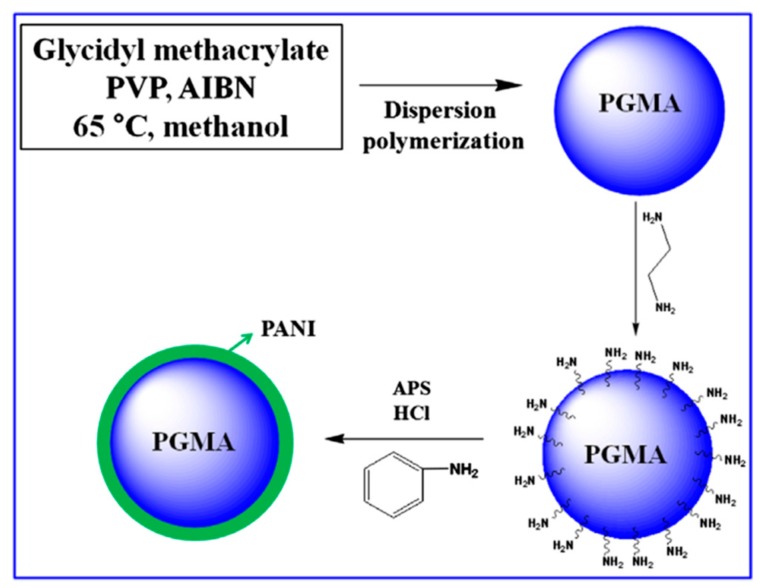
Schematic diagram of the experimental route to synthesize PGMA/PANI particles (Zhang et al. [123], Copyright Elsevier, 2013).

As mentioned above, in the synthesis of PANI/PMMA, poly(glycidyl methacrylate) (PGMA) was synthesized on the surface of PMMA to graft the aniline functionality. Zhang et al. [123] reported the facile and fast synthesis of core-shell structured PGMA/PANI microspheres and applied these microspheres to ER materials. Scheme 6 presents a schematic diagram of the synthetic procedure. Monodispersed PGMA particles were synthesized by the dispersion polymerization of GMA in the methanol medium with PVP as a stabilizer and AIBN as an initiator. Amine groups were then grafted onto the surface of PGMA through an epoxy-amine reaction between the ethylenediamine and glycidyl groups of PGMA. Finally, the aniline monomers adhered to the surface of PGMA through the covalent bonds formed between the aniline and amine group-modified PGMA via amine-amine functional groups and polymerized by chemical oxidative polymerization with APS as an initiator.

**Figure 6:**
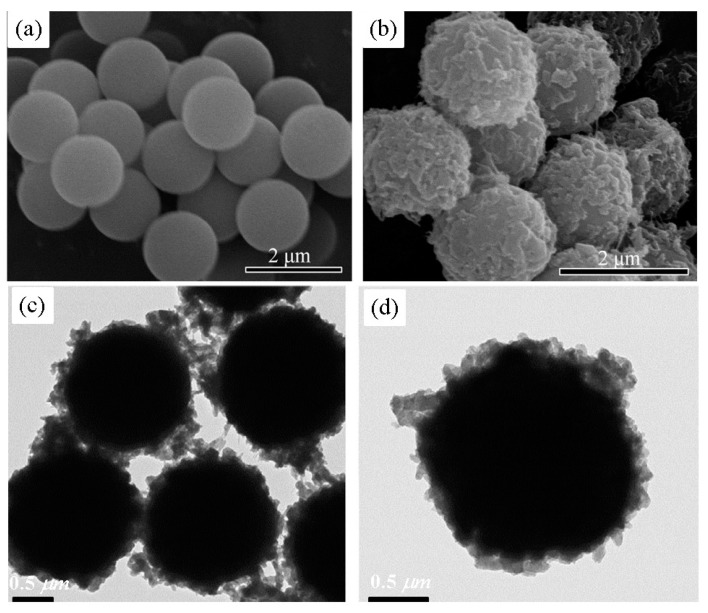
SEM images of (**a**) PGMA microspheres, (**b**) PGMA/PANI particles. In addition, (**c**,**d**) TEM images of PGMA/PANI particles. (Zhang et al. [123], Copyright Elsevier, 2013).

Figure 6 shows SEM and TEM images of PGMA and PGMA/PANI particles. The PGMA are spherical and monodispersed with a diameter of approximately 1.6 μm. After coating PANI, the surface became rougher than pure PGMA. TEM showed that the coated PANI layers were not uniform.

#### 4.2.3. Polystyrene

**Scheme 7:**
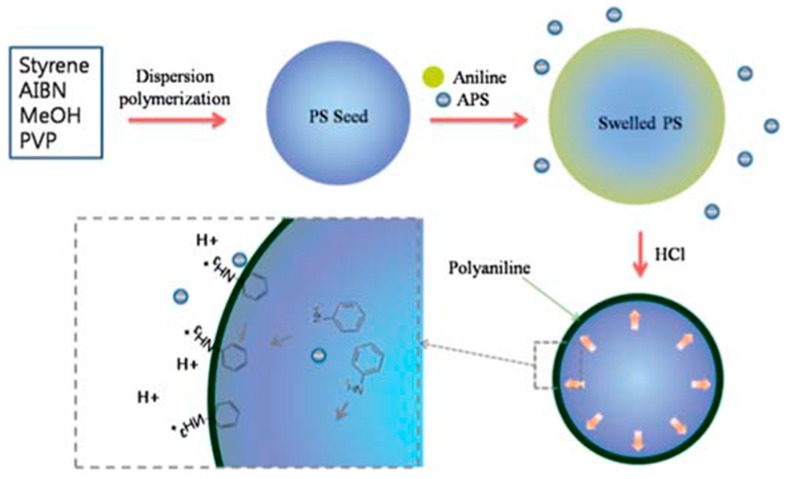
Schematic diagram of PS/PANI microsphere preparation. (Liu et al. [126], Copyright The Royal Society of Chemistry, 2011).

Core-shell structured PS/PANI microspheres are also considered a promising ER materials because spherical and monodisperse polystyrene (PS) microspheres are easy to prepare [124]. In contrast to coating PANI on the PMMA and PGMA surface, the process of coating PANI onto the PS is much simpler. Kim et al. [125] and Liu et al. [126] coated PANI on the surface of PS by the diffusion and polymerization of aniline at the interface without a surfactant or surface modification of PS. Scheme 7 presents a graphical diagram of the synthetic process.

The monodispersed PS microspheres with a diameter of 1–2 μm were prepared using a facile dispersion polymerization method in a methanol medium with PVP as a stabilizer and AIBN as an initiator. When PS microspheres, aniline monomers, and APS were dispersed in DI-water, the aniline monomers will be adsorbed on the PS through π-π* stacking interactions. After the addition of HCl, the aniline monomers begin to polymerize and form a PANI shell on the PS surface.

**Scheme 8:**
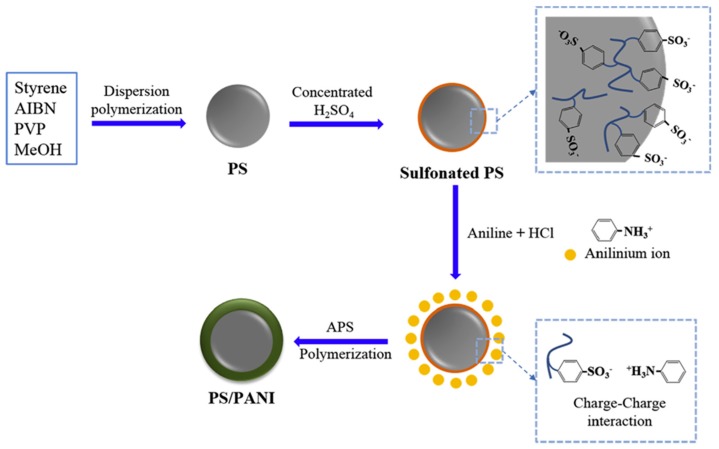
Schematic diagram of the experimental route to synthesize PS/PANI particles. (Piao et al. [127], Copyright Elsevier, 2017).

Piao et al. [127] modified the surface of PS with concentrated sulfuric acid (H_2_SO_4_) before coating with PANI, allowing the aniline monomers to adsorb better on the PS surface through charge-charge interactions to achieve a better PANI shell. Scheme 8 shows a graphical diagram of their experimental route. The PS microspheres were also prepared by a dispersion polymerization method, while in this study, the diameter of the PS particles were approximately 360 nm. After modification with concentrated H_2_SO_4_, the surface of the PS particles were negatively charged, and the aniline monomers protonated by HCl were positively charged. Therefore, the aniline monomers will be adsorbed on the surface of the PS particles through charge-charge interactions, and polymerization begins after adding the APS initiator.

**Figure 7:**
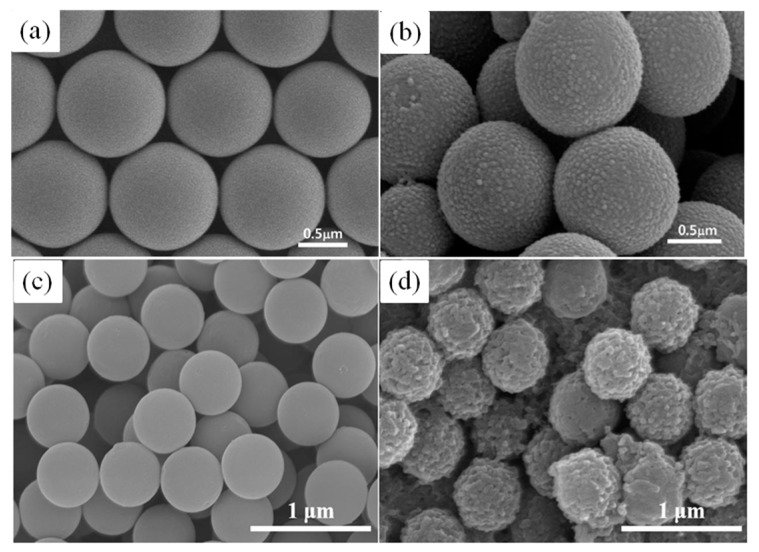
SEM images of PS and PS/PANI synthesized by (**a**,**b**) Liu et al. [126] (Copyright The Royal Society of Chemistry, 2011) and (**c**,**d**) Piao et al. [127] (Copyright Elsevier, 2017).

Figure 7 compares SEM images of PS and PS/PANI synthesized by (a,b) Liu et al. [126] and (c,d) Piao et al. [127], clearly showing that the PS particles prepared by Piao et al. [127] are smaller, which may be related to the molecular weight of PVP and the amount of initiator during the synthesis process. In addition, the synthesized PS/PANI surfaces became rougher with increasing PANI shell thickness of 70 nm from the TEM images while the thickness of the coated PANI shell from Liu et al. [126] was 50 nm, indicating that surface modification with sulfuric acid is advantageous to form a better PANI shell on the PS core.

**Figure 8:**
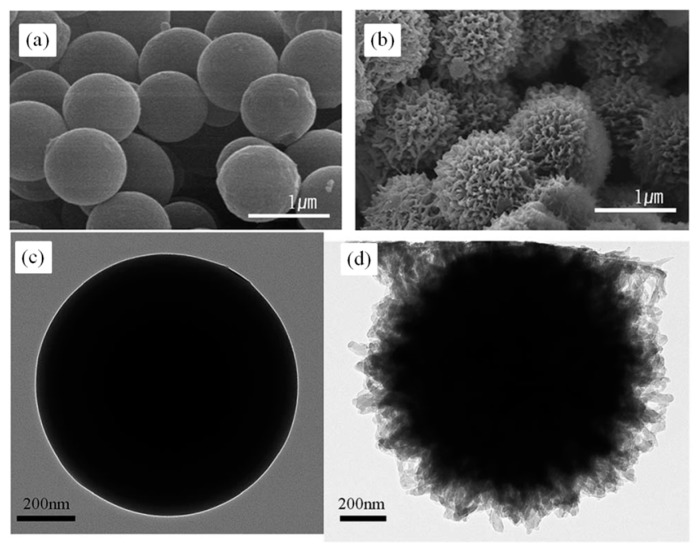
SEM (**a**,**b**) and TEM (**c**,**d**) images of polystyrene seed particle and urchin-like PS/PANI particle. (Kim et al. [128], Copyright The Royal Society of Chemistry, 2015).

On the other hand, for shell material with different morphologies, Kim et al. [128] synthesized interesting sea urchin-like core-shell-type PS/PANI particles by a seeded swelling polymerization process using ferric nitrate as an oxidant instead of the usual APS, without even using HCl. The monodispersed PS seed was prepared by the dispersion polymerization of styrene in an isopropyl alcohol aqueous solution. The PS seeds and aniline monomers were then dispersed in water, and aniline monomers were attached on the surface of PS through the π-π* stacking interactions. The chemical oxidative polymerization began after adding ferric nitrate, and finally formed urchin-like core-shell PS/PANI particles. Figure 8 shows SEM and TEM images of the PS and urchin-like core-shell PS/PANI particles synthesized [128]. The mean diameter of PS and urchin-like PS/PANI particles were approximately 1 and 1.3 μm, respectively. After coating with PANI, the surface of the particles became uneven, and the morphology of the PANI shell was significantly different from that synthesized with APS as an oxidant.

Overall, the advantage of using polymeric cores is their proper control of shape and size while their ER efficiency is limited especially for non-functional commodity polymers such as PMMA and PS.

## 5. Electrorheological Characteristics

**Scheme 9:**
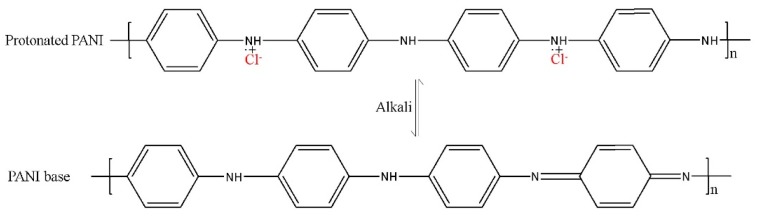
Schematic diagram of the conversion process between protonated PANI (HCl as an example) and PANI base by treating with alkali.

ER fluids are typically prepared by dispersing electro-responsive particles in non-conducting oils, such as silicone oil or mineral oil, while the dispersed particle need to be semi-conducting. In the case of PANI, however, which is generally synthesized by an oxidative polymerization process in an acid environment with its protonation, the pristine PANI appears to be the highly electrically conductive emeraldine salt form, which is likely to cause electric breakdown during the ER measurements. On the other hand, protonated PANI can be converted to a lowly electrically conductive PANI base by a treatment with alkali [129,130]. Therefore, before applying the PANI-coated core-shell microspheres to the ER fluids, a de-doping process is generally necessary to reduce the electrical conductivity of the particles in the semi-conducting regime to prevent electric breakdown at high electric field strengths. Scheme 9 illustrates the conversion process between protonated PANI (HCl as an example) and PANI base.

For ER fluids, among the various rheological characteristics, the shear stress and dynamic yield stress are two very important properties, which will be discussed briefly for PANI-coated core-shell microsphere-based ER fluids.

### 5.1. Shear Stress

The shear stress of the ER fluids is generally measured from a controlled shear rate (CSR) test mode under various electric field strengths, and increases linearly with increasing shear rate in the absence of an electric field, indicating Newtonian fluid-like characteristics. On the other hand, upon the application of an electric field, ER fluids exhibit Bingham fluid-like properties with a significant yield stress [131,132]. The Bingham model, a well-known rheological equation of state used to explain the shear stress characteristics of suspensions, has also been adopted to ER fluids under an electric field. The model has two flow regimes: a pre-yield region with a stable shear stress value for shear stresses lower than the dynamic yield stress ($\tau_{y}$), and a post-yield region for shear stresses beyond the $\tau_{y}$, showing Newtonian fluid behavior [133]. The Bingham model can be expressed as follows:

(3a) $$\tau =\tau_{y}+\eta_{pl}\dot{\gamma },\;\tau \geq \tau_{y},$$

(3b) $$\dot{\gamma }=0,\;\tau <\tau_{y}$$

where the $\dot{\gamma }$ is the shear rate; $\eta_{pl}$ is the plastic viscosity, which is generally regarded as a constant value for the same fluid; and *τ_y_* is the yield stress, which is a function of the electric field strengths.

**Figure 9:**
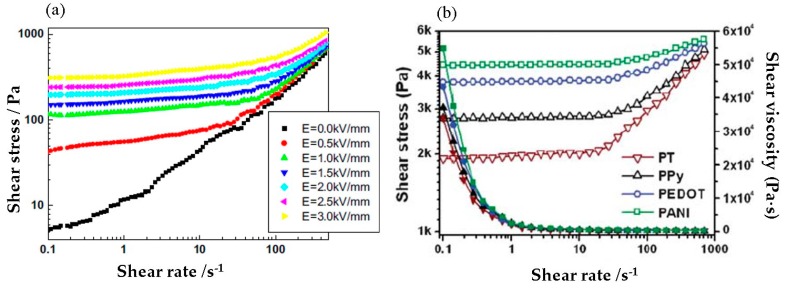
(**a**) Shear stress curves for Fe_2_O_3_/PANI-based ER fluid (20 wt % in silicone oil) at various electric field strengths (Tian et al. [98], Copyright Elsevier, 2016) and (**b**) shear stress (open symbols) and shear viscosity (closed symbols) curves for various conducting polymer coated SiO_2_ core-shell nanospheres-based ER fluids (30 vol % in silicon oil) under 3 kV/mm of electric field (Hong et al. [85], Copyright The Royal Society of Chemistry, 2010).

Figure 9 shows the shear stress versus shear rate for Fe_2_O_3_/PANI and various conducting polymer (PT, PPy, PEDOT and PANI)-coated SiO_2_ core-shell nanosphere-based ER fluids on a log-log scale. As shown in Figure 9a, in the absence of an electric field, the shear stress of Fe_2_O_3_/PANI-based ER fluid increases linearly with increasing shear rate, exhibiting Newtonian fluid properties. In the case of an applied electric field, the shear stress remains stable over a low shear rate range at each electric field intensity, and when the shear rate is relatively high, the shear stress begins to increase, which is consistent with the Bingham model. Figure 9b shows the shear stress curves of core-shell structured SiO_2_/PT, SiO_2_/PPy, SiO_2_/PEDOT, and SiO_2_/PANI-based ER fluids under an electric field strength of 3 kV/mm. All curves showed characteristics consistent with the Bingham model and the SiO_2_/PANI-based ER fluid has the highest shear stress, indicating that the strongest chain-like structure may be formed between the SiO_2_/PANI particles under the same magnetic field strength.

**Figure 10:**
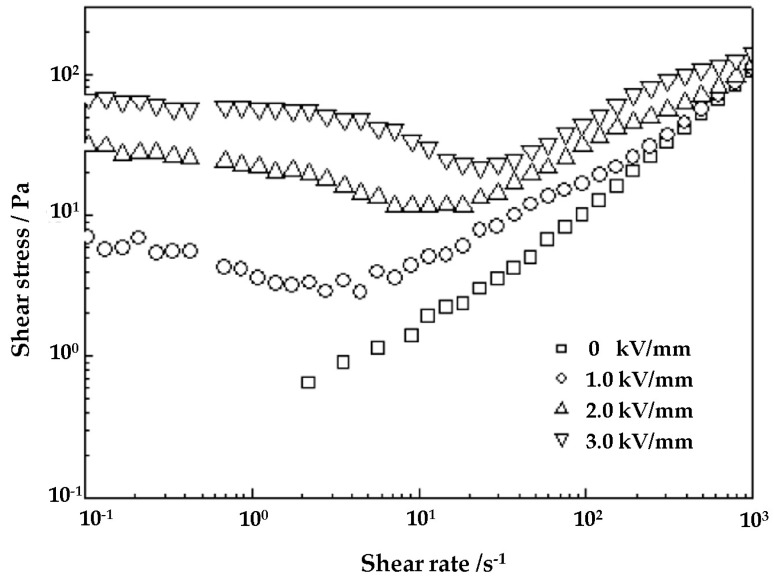
Shear stress versus the shear rate for the PMMA/PANI-based ER fluid (10 vol % in silicone oil) under various electric field strengths. (Lee et al. [121], Copyright Elsevier, 2005).

In many cases, however, the simple Bingham model with two parameters cannot match the shear stress curve exactly. As shown in Figure 10, the shear stress curves of the PMMA/PANI-based ER fluids show a decreasing trend in the low shear rate range, which is inconsistent with the Bingham model, in which the shear stress at a low shear rate region remains stable. Therefore, further modified rheological equations of state were presented to explain such ER characteristics, known as the Cho-Choi-Jhon (CCJ) model [134,135,136], which can be expressed as

(4) $$\tau =\frac{\tau_{y}}{1+{\left(t_{2}\dot{\gamma }\right)}^{\alpha }}+\eta_{\infty }[1+\frac{1}{{\left(t_{3}\dot{\gamma }\right)}^{\beta }}]$$

where $\tau_{y}$ is the dynamic yield stress; *α* is related to the decrease of shear stress in the low shear rate region; *t*_2_ and *t*_3_ are time parameters; $\eta_{\infty }$ is the shear viscosity at the infinite shear rate; the exponent, *β*, is related to the increase in shear stress in the high shear rate region with its range of 0<β≤1. This model with six parameters can explain better the shear stress curve with decreasing tendency in a low shear rate region and provide a more reliable dynamic yield stress.

**Figure 11:**
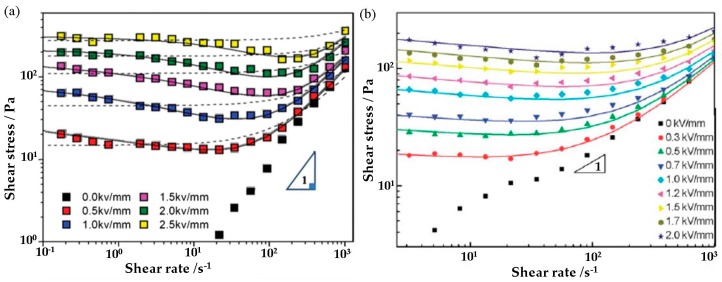
Shear stress versus shear rate for (**a**) PMMA/PANI-based ER fluid (20 wt % in silicone oil), the dashed lines were fitted using a conventional Bingham model, the solid lines were fitted via a CCJ model (Fang et al. [122], Copyright The Royal Society of Chemistry, 2011) and (**b**) PS/PANI-based ER fluid (10 vol % in silicone oil), the solid lines are fitted via a CCJ model (Liu et al. [126], Copyright The Royal Society of Chemistry, 2011).

As shown in Figure 11a, Fang et al. [122] fitted the shear stress curve for PMMA/PANI-based ER fluid with both Bingham model and CCJ model, and the results showed that the Bingham model was unable to fully describe the curve, while the CCJ model fitted the data well. Liu et al. [126] also adopted the CCJ model to fit the shear stress curve for the PS/PANI-based ER fluid.

**Figure 12:**
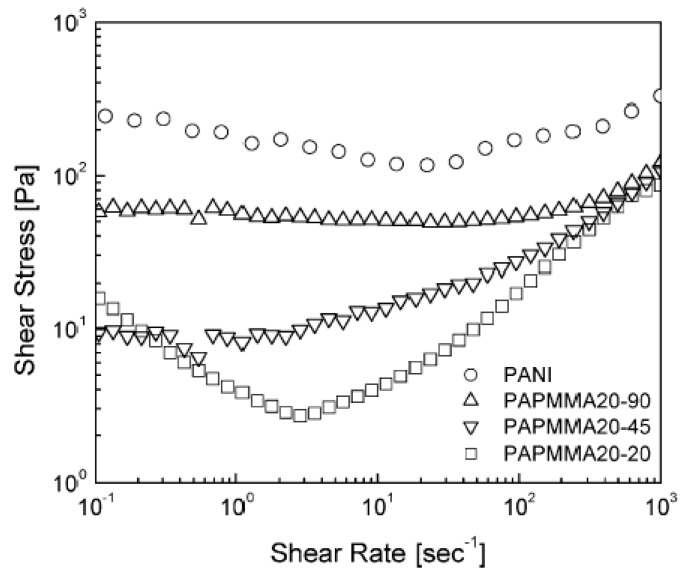
Shear stress as a function of the shear rate for pure PANI and core-shell structured PMMA/PANI with a core size of 2 μm (PAPMMA20-20), 4.5 μm (PAPMMA20-45), and 9 μm (PAPMMA20-90)-based ER fluids (10 vol % in silicone oil) at an electric field strength of 3 kV/mm. (Cho et al. [57], Copyright American Chemical Society, 2003).

**Scheme 10:**
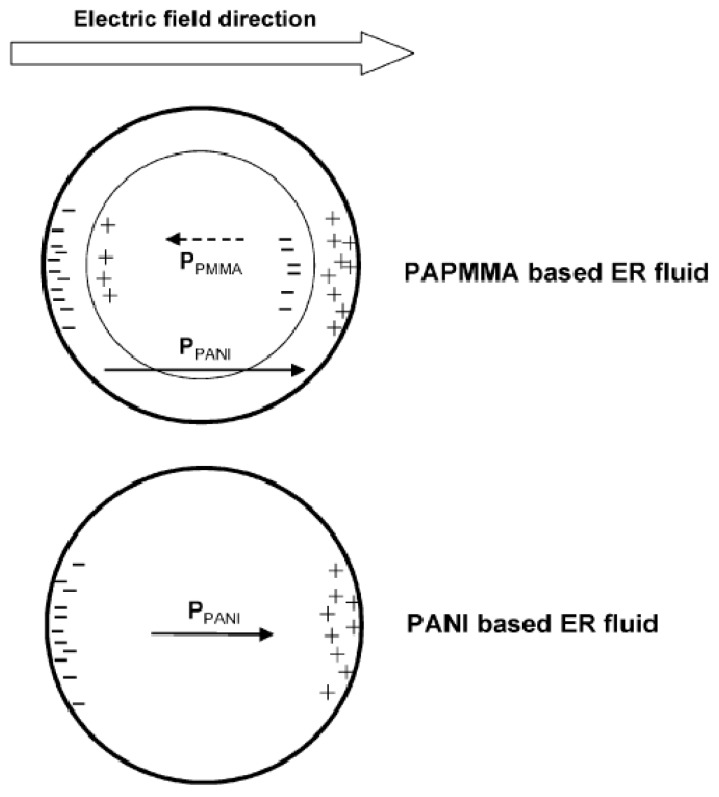
Possible scheme of the polarization behavior for PMMA/PANI and PANI particles under an electric field. (Cho et al. [57], Copyright American Chemical Society, 2003).

The shear stress behavior of the ER fluid based on different types of PANI-coated core-shell-type microspheres may be in accordance with the Bingham model or the CCJ model, and the particle size also has an important effect on the behavior of the shear stress curve. Figure 12 presents the shear stress curves of pure PANI and core-shell structured PMMA/PANI particle-based ER fluids with different core sizes (2, 4.5 and 9 μm) under an electric field strength of 3 kV/mm. The shear stress of the ER fluids based on PMMA/PANI with a core size of 4.5 and 9 μm exhibited a relatively stable value at low shear rates, which is consistent with the Bingham model. On the other hand, for an ER fluid based on PMMA/PANI particles with a core size of 2 μm, the shear stress exhibited a significant downward trend at a low shear rate range, which is more consistent with the CCJ model. In addition, the shear stress of the pure PANI-based ER fluid is higher than that of the PMMA/PANI-based ER fluid, which is probably because with pure PANI, polarization occurs throughout the particle, but for PMMA/PANI particles, polarization only occurs in the PANI shell, and even the PMMA core will be polarized by the movement of electrons in the PANI shell, which reduces the electrostatic interactions among the PMMA/PANI particles. Scheme 10 shows a proposed schematic diagram of polarization behavior of PMMA/PANI and PANI particles under an electric field.

Under an applied electric field, the particles in the ER fluid are polarized to form a chain-like structure along the direction of the electric field via electrostatic interactions. On the other hand, the chain-like structure will be interrupted by the shear flow in the direction perpendicular to the electric field and reorganized by electrostatic interactions. In other words, there is competition for hydrodynamic interaction and electrostatic interactions in the ER fluid system [137]. The behavior of the shear stress under an applied electric field is dependent on the balance between the hydrodynamic and electrostatic interactions. For the Bingham model, the shear stress is stable in the low shear rate range because the chain-like structure interrupted by the shear flow can be reorganized rapidly by the electric field, but as the shear rate increases to a certain extent, the dynamic interaction dominates, the structure is severely destroyed, and the shear stress increases with increasing shear rate, which exhibits fluid-like behavior. In contrast, the CCJ model explains the special behavior of shear stress decreasing with increasing shear rate in the low shear rate range, which may be because the chain-like structure reorganized by the electric field is incomplete compared to that before the application of shear flow. In other words, the rate of reorganization of the chain-like structure cannot match the rate of destruction. On the other hand, it can be also noted that the PANI has been encapsulated with melamine-formaldehyde resin for the ER study which is the inverse case of the majority of the PANI shell in this review [138].

### 5.2. Yield Stress ($\tau_{y}$)

**Figure 13:**
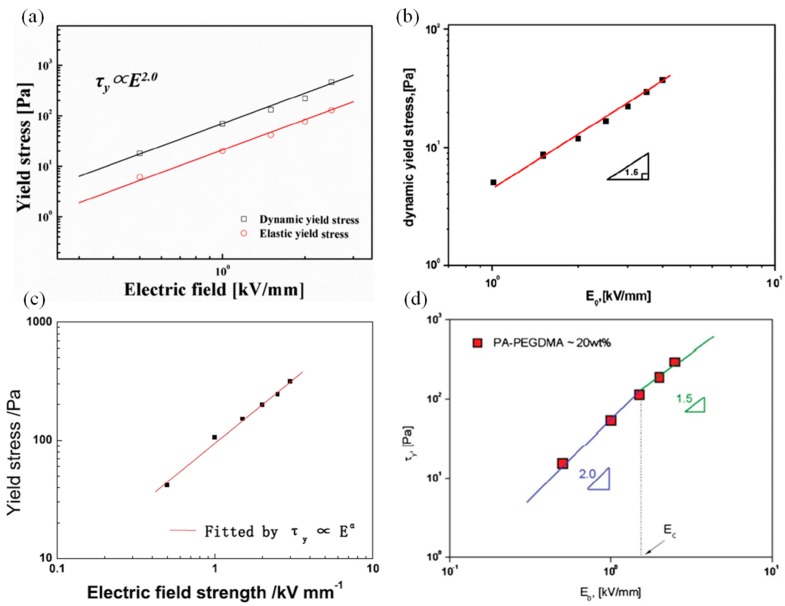
Yield stress versus electric field strength for (**a**) PS/PANI (Piao et al. [127], Copyright Elsevier, 2017); (**b**) PGMA/PANI (Zhang et al. [123], Copyright Elsevier, 2013); (**c**) Fe_2_O_3_/PANI (Tian et al. [98], Copyright Elsevier, 2016) and (**d**) PMMA/PANI (Fang et al. [122], Copyright The Royal Society of Chemistry, 2011)-based ER fluids.

Among the different yield stresses dependent on its measurement, the dynamic yield stress is the minimum stress that can continuously interrupt the chain-like structure among the particles, which is usually evaluated by extrapolating the shear stress to a zero shear rate limit from the shear stress flow curve. The relationship between the yield stress and electric field strength can be expressed as

(5) $$\tau_{y}\propto E^{\alpha }$$

where $\tau_{y}$ is the yield stress and *E* represents the electric field strength; the value of the index *α* is usually between 1.0 and 2.0. When *α* = 2.0, this equation corresponds to a polarization model, while *α* = 1.5 corresponds to a conduction model. Figure 13a–d show the dynamic yield stress versus electric field strength on a log-log scale for PS/PANI, PGMA/PANI, Fe_2_O_3_/PANI, and PMMA/PANI-based ER fluids, respectively. PS/PANI-based ER fluid (Figure 13a) shows a polarization model with slope equal to 2.0 while the PGMA/PANI-based ER fluid (Figure 13b) shows a conduction model with a slope equal to 1.5. The particle concentration, particle morphology and size, electric field strength, and dielectric properties of the ER fluid etc. are also known to affect the dynamic yield stress; so sometimes, the relationship between the dynamic yield stress and electric field is not fully consistent with the conductive model or polarization model [139]. As shown in Figure 13c, the slope of the Fe_2_O_3_/PANI-based ER fluid is 1.97, which is not consistent with the slope of the polarization model or the conduction model, but it can be considered an approximate polarization model.

Figure 13d shows the dynamic yield stress results of PMMA/PANI-based ER fluid, in which the slope changed from 2.0 to 1.5 at the electric field strength turning point, called the critical electric field strength (*E_c_*). In view of this situation, Choi et al. [140] introduced the universal yield stress equation, which correlates the yield stress with a wide range of electric field strengths, as shown in Equation (6):

(6) $$\tau_{y}\left(E_{0}\right)=\alpha E_{0}^{2}\left(\frac{\tan h\sqrt{E_{0}/E_{c}}}{\sqrt{E_{0}/E_{c}}}\right)$$

where *α* depends on the particle concentration and dielectric properties of the ER fluid and this equation was normalized by *E_c_* and $\tau_{y}\left(E_{c}\right)=0.762\alpha E_{c}^{2}$ to Equation (7):

(7) $$\hat{\tau }=1.313{\hat{E}}^{3/2}\tan h\sqrt{\hat{E}}$$

where $\hat{E}\equiv E_{0}/E_{c}$ and $\hat{\tau }\equiv \tau_{y}\left(E_{0}\right)/\tau_{y}\left(E_{c}\right)$.

**Figure 14:**
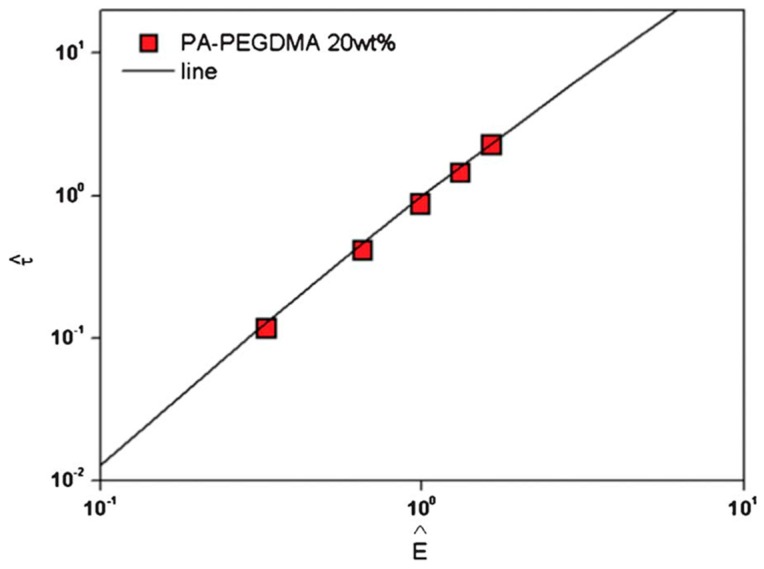
$\hat{\tau }$ versus $\hat{E}$ for PMMA/PANI-based ER fluids. The solid line was drawn using Equation (7). (Fang et al. [122], Copyright The Royal Society of Chemistry, 2011).

As shown in Figure 14, the data obtained from Figure 13d collapsed onto a single curve by Equation (7). In addition, Choi et al. [141] further introduced the parameter *b* to obtain a better linear relationship between the dynamic yield stress and electric field, as follows:

(8) $$\tau_{y}\left(E_{0}\right)=\alpha E_{0}^{2}\left(\frac{\tan h{\left(E_{0}/E_{0}\right)}^{0.5+b}}{{\left(E_{0}/E_{c}\right)}^{0.5+b}}\right)$$

after rescaling $\hat{\tau }$ and $\hat{E}$ with $\hat{\hat{\tau }}=\hat{\tau }{\hat{E}}^{4b}$ and $\hat{\hat{E}}={\hat{E}}^{1+2b}$, Equation (9) can be derived:

(9) $$\hat{\hat{\tau }}=1.313{\hat{\hat{E}}}^{1.5}\tan h{\hat{\hat{E}}}^{0.5}$$

This equation proved to be suitable for many ER fluids or even MR fluids [141,142].

## 6. Dielectric Analysis

**Figure 15:**
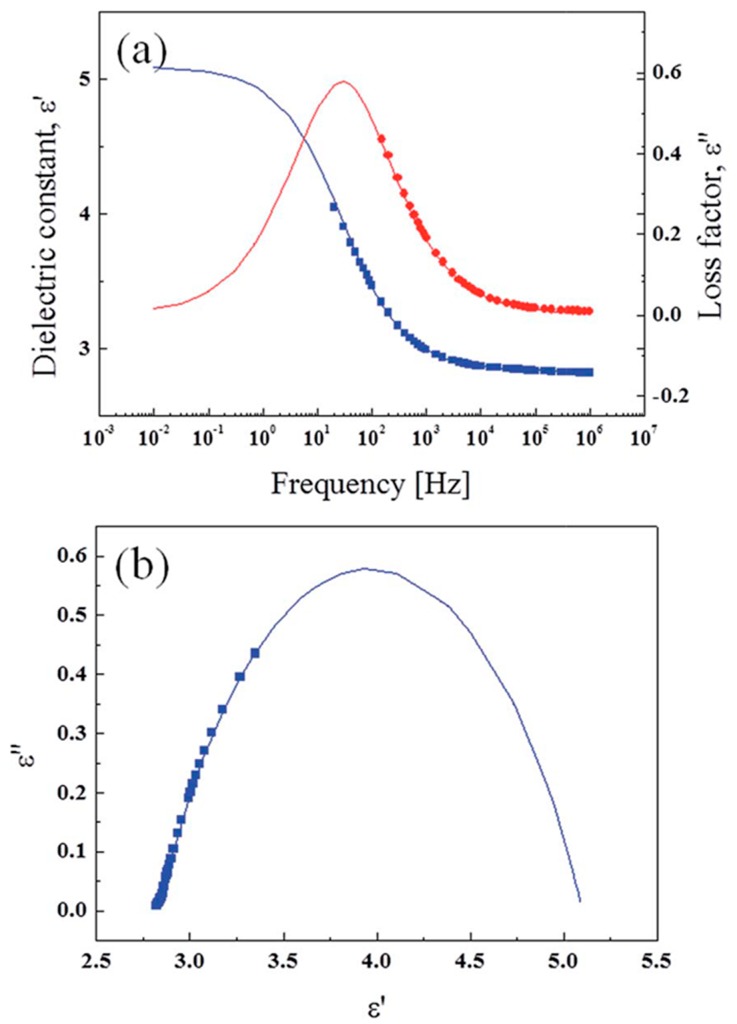
Dielectric properties for PS/PANI-based ER fluid: (**a**) Dielectric constant (blue square) and loss factor (red circle) as a function of the frequency; (**b**) Loss factor as a function of the dielectric constant. The solid lines are drawn using Equation (10). (Kim et al. [128], Copyright The Royal Society of Chemistry, 2015).

Among the many mechanisms proposed for ER fluids, the polarization model is well recognized by many researchers, and the dielectric properties of ER fluids, including the dielectric constant ($\varepsilon^{\prime }$) and dielectric loss factor ($\varepsilon^{\prime \prime }$), are believed to be closely related to the ER properties. A well-known Cole-Cole formula [143] is commonly used to relate the dielectric properties and ER properties, which can be expressed as

(10) $$\varepsilon^{*}=\varepsilon^{\prime }-\varepsilon^{\prime \prime }{=\varepsilon }_{\infty }+\frac{\varepsilon_{0}-\varepsilon_{\infty }}{1+{\left(i\omega \lambda \right)}^{1-\alpha }},\;0\leq \alpha \leq 1$$

where $\varepsilon^{*}$ is the complex dielectric constant; $\varepsilon_{0}$ and $\varepsilon_{\infty }$ are the dielectric constants at zero frequency and infinite frequency, respectively; and $\Delta \varepsilon =\varepsilon_{0}-\varepsilon_{\infty }$ is related to the achievable polarizability of the ER fluid. $\lambda =1/2\pi f_{max}$ is the dielectric relaxation time of the interfacial polarization of the ER fluid, where the $f_{max}$ is the frequency at which the dielectric loss factor is a maximum. A large Δε and short λ have positive effects on the ER performance. Figure 15 shows the dielectric properties for a PS/PANI-based ER fluid. The data matched well with Equation (10), and the parameters are listed in Table 1. According to the fitting results, the large Δ*ε* and short λ indicate that the ER fluid based on PS/PANI exhibits excellent ER characteristics, as evidenced by the ER test results.

**Figure 16:**
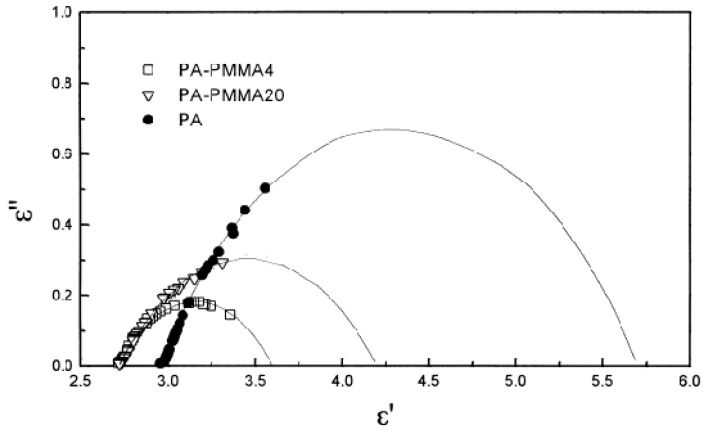
Loss factor for ER fluids based on PANI (PA) and PMMA/PANI with PANI shell thickness of 11 (PA-PMMA4) and 52 nm (PA-PMMA20) as a function of the dielectric constant. The solid lines were drawn using Equation (10). (Cho et al. [120], Copyright Springer Nature, 2002).

Cho et al. [120] compared the parameters of Cole-Cole equation for different ER fluids. Figure 16 shows the Cole-Cole fitting results for ER fluids based on PANI (PA) and PMMA/PANI with a PANI shell thickness of 11 (PA-PMMA4) and 52 nm (PA-PMMA20). Table 2 lists the fitted parameters from Equation (10). As shown in Table 2, the ER fluid based on PMMA/PANI showed a lower Δε than pure PANIbecause the insulating PMMA core counteracts the interfacial polarization. As expected, the value of Δε for the ER fluid based on PMMA/PANI with a PANI shell thickness of 52 nm is higher than that with a PANI shell thickness of 11 nm. On the other hand, there was no obvious rule of λ in this study, which may be because λ is also dependent on other factors, such as the particle size, etc. Therefore, more extensive research will be needed to summarize the relationship between the dielectric and ER properties of ER fluids and the factors that influence the dielectric properties.

## 7. Conclusions

This paper reviewed various electro-stimuli responsive PANI-coated core-shell-type microspheres, which were divided into two categories: one with the inorganic materials as the core, including SiO_2_, Fe_3_O_4_, Fe_2_O_3_, and TiO_2_; and the other using the polymeric materials as the core, including PMMA, PS, and PGMA. Table 3 the summarize various experimental parameters including the synthetic method, morphology, particle size and the thickness of PANI shell, in which the thickness of the PANI shell ranged from 8 nm to 485 nm, depending on the synthetic method, the amount of aniline input, and the shape and size of the cores. Because high electrical conductivity of the PANI easily leads to electric breakdown in ER fluids during test, its de-doping process is usually necessary. Therefore based on this fact, too thick PANI shell could more likely lead to electrical breakdown or make its de-doping process more complicated, while too thin PANI shell may reduce ER performance. Therefore a proper PANI thickness needs to be considered.

The use of an inorganic core and PANI shell to form core-shell structured microspheres is a valuable method for developing excellent hybrid materials, combining the advantages of high hardness, high mechanical strength, and thermal resistance of the inorganic core and the excellent electrical properties of the PANI shell. On the other hand, the density of the inorganic core is usually higher than PANI, making the density of inorganic-PANI microspheres higher than pure PANI, thereby reducing the dispersion stability of the particles in ER fluids. The polymeric core generally have a low density compared to inorganic cores, but the synthetic process of polymeric-PANI microspheres is more complex, such as the need for surface modification for polymeric core.

In the absence of an external electric field, the shear stress behavior of ER fluids based on different types of PANI-coated core-shell typed microspheres exhibits Newtonian fluid-like behavior. Under electric fields, however, the shear stress can reach hundreds or even thousands of times higher than that in the absence of an electric field and the shear stress curves are in accordance with the Bingham or CCJ models. On the other hand, the shear stress of the pure PANI-based ER fluid was higher than that if the PANI-coated core-shell particle-based ER fluid, probably because for pure PANI, polarization occurs throughout the entire particle, whereas for the PANI coated core-shell-type microspheres, polarization only occurs in the PANI shell. Even the core will be polarized by the movement of electrons in the PANI shell and reduce the electrostatic interactions between the PANI-coated core-shell particles, leading to lower shear stress. In addition, the particle size also has an important effect on the behavior of the shear stress curve.

The relationship between the dynamic yield stress for the PANI-coated core-shell typed microsphere-based ER fluid and electric field strength can be expressed by a power law equation, in which the exponent is usually between 1.0 and 2.0. When the exponent equal to 2.0, the relationship between dynamic yield stress and electric field strength corresponds to a polarization model, whereas it corresponds to a conduction model when the exponent equal to 1.5. The exponent is also dependent on the particle concentration, particle morphology, and size; the applied electric field strength; and dielectric properties of the ER fluid. Furthermore, in some cases, where the relationship between the dynamic yield stress and the electric field strength changes from the polarization model to the conductivity model, a universal yield stress equation was proven that can correlate linearly the dynamic yield stress with a wide range of electric field strengths.

The dielectric properties are believed to be closely related to the ER properties, and a Cole-Cole formula is generally used to explain this relationship. Among the many parameters of this formula, the difference dielectric constants at zero frequency and infinity (Δε) and the dielectric relaxation time of the interfacial polarization (λ) are considered to have a significant effect on the ER characteristics. On the other hand, the relationship between the dielectric and ER properties of ER fluids and the factors influencing the dielectric properties need to be examined in a future study.

Several mechanisms for ER fluids were proposed, in which the polarization mechanism and conduction model are considered to be suitable explanations for the ER characteristics of PANI-coated core-shell typed microsphere-based ER fluids. Nevertheless, different models for theoretical and experimental research are still needed. Furthermore, in addition to many PANI-coated core-shell typed materials which have been already adopted as for ER materials, the PANI could be replaced with other excellent conducting polymers such as poly(diphenylamine) [144], PPy, and PANI-copolymer even though their reactivity might not be as efficient as PANI.

## Figures and Tables

**Table 1:** Parameters in Equation (10) for a PS/PANI-based ER fluid. (Reprinted from [128], Copyright The Royal Society of Chemistry, 2015).

$\varepsilon_{0}$	*ε*_∞_	Δε	*α*	λ
5.1	2.83	2.27	0.4	0.0057

**Table 2:** Parameters in Equation (10) in Figure 16. (Reprinted from [120], Copyright Springer Nature, 2002).

ER Fluids	$\varepsilon_{0}$	$\varepsilon_{\infty }$	Δε	λ	*α*
PA-PMMA4	3.6	2.72	0.88	0.002	0.50
PA-PMMA20	4.2	2.72	1.48	0.020	0.50
PA	5.7	2.96	2.74	0.040	0.41

**Table 3:** Summary of PANI coated core-shell typed microspheres reviewed in this work.

	Core	Synthetic Method	Morphology	Size	Thickness of PANI Shell
Trlica et al. [83]	SiO_2_	in situ polymerization	spherical	15 μm	-
Hong et al. [85]	SiO_2_	in situ polymerization	spherical	<30 nm	<8 nm
Park et al. [86]	SiO_2_	in situ polymerization	spherical	1 μm	50 nm
Tian et al. [98]	Fe_2_O_3_	in situ polymerization	flower-like	1–1.5 μm	_
Sim et al. [109]	Fe_3_O_4_	in situ polymerization	spherical	0.7–1.0 μm	100 nm
Wang et al. [116]	TiO_2_	in situ polymerization	spherical	400–500 nm	50 nm
Tian et al. [117]	TiO_2_	in situ polymerization	peanut-like	-	-
Cho et al. [57]	PMMA	in situ polymerization	spherical	2 μm 4.5 μm 9 μm	51.7 nm 130.7 nm 261.4 nm
Cho et al. [120]	PMMA	in situ polymerization	spherical	2 μm	52 nm
Lee et al. [121]	PMMA	grafting polymerization	spherical	10 μm	-
Fang et al. [122]	PMMA	grafting polymerization	spherical	10.6 μm	485 nm
Zhang et al. [123]	PGMA	grafting polymerization	spherical	1.6 μm	120 nm
Liu et al. [126]	PS	diffusion-interface polymerization	spherical	1–2 μm	50 nm
Piao et al. [127]	PS	in situ polymerization	spherical	50 nm	70 nm
Kim et al. [128]	PS	seeded swelling emulsion polymerization	urchin-like	1.3 μm	300 nm

## References

[1] Marins J.A., Soares B.G. (2017). Ionic liquid-based organically modified silica for the development of new electrorheological fluids. Colloids Surf. A Physicochem. Eng. Asp..

[2] Cabuk M. (2017). Electrorheological response of mesoporous expanded perlite particles. Mesoporous Mesoporous Mater..

[3] Do T.G., Lee H.J., Ko Y.G., Chun Y.S., Choi U.S., Kim C.H. (2017). Influence of amine- and sulfonate-functional groups on electrorheological behavior of polyacrylonitrile dispersed suspension. Colloids Surf. A Physicochem. Eng. Asp..

[4] Zhang Z.L., Zhang Z.G., Hao B.N., Zhang H.Y., Wang M., Liu Y.D. (2017). Fabrication of imidazolium-based poly(ionic liquid) microspheres and their electrorheological responses. J. Mater. Sci..

[5] Kamelreiter M., Kemmetmüller W., Kugi A. (2012). Digitally controlled electrorheological valves and their application in vehicle dampers. Mechatronics.

[6] Jose R.R., Elia R., Tien L.W., Kaplan D.L. (2014). Electroresponsive aqueous silk protein as “smart” mechanical damping fluid. ACS Appl. Mater. Interface.

[7] Tan K.P., Stanway R., Bullough W.A. (2006). Dynamic velocity response of an electro-rheological (ER) clutch for robotic applications. Mech. Adv. Mater. Struct..

[8] Choi S.B., Yook J.Y., Choi M.K., Nguyen Q.H., Lee Y.S., Han M.S. (2007). Speed control of DC motor using electrorheological brake system. J. Intell. Mater. Syst. Struct..

[9] Furusho J., Sakaguchi M., Takesue N., Koyanagi K. (2002). Development of ER brake and its application to passive force display. J. Intell. Mater. Syst. Struct..

[10] Su J.S., Cheng H.B., Wen Y.F., Feng Y.P., Tam H.Y. (2016). Investigation into the mechanism for ultra smooth electrorheological finishing using wheel-like finishing tool. J. Mater. Process. Technol..

[11] Pandey A.K., Pandey P.C., Agrawal N.R., Das I. (2018). Synthesis and characterization of dendritic polypyrrole silver nanocomposite and its application as a new urea biosensor. J. Appl. Polym. Sci..

[12] Sriwichai S., Janmanee R., Phanichphant S., Shinbo K., Kato K., Kaneko F., Yamamoto T., Baba A. (2018). Development of an electrochemical-surface plasmon dual biosensor based on carboxylated conducting polymer thin films. J. Appl. Polym. Sci..

[13] Jo W.G., Khan M., Tan L.S., Jeong H.S., Lee S.H., Park S.Y. (2017). Polypyrrole nanocomposite with water-dispersible graphene. Macromol. Res..

[14] Zhang J., Zeng B.Z., Zhao F.Q. (2018). Fabrication of bi-monomer copolymer of pyrrole-indole for highly efficient solid phase microextraction of benzene derivatives. Talanta.

[15] Suganya N., Jaisankar V., Sivakumar E.K.T. (2017). Conducting polymeric hydrogel electrolyte based on carboxymethylcellulose and polyacrylamide/polyaniline for supercapacitor applications. Int. J. Nanosci..

[16] Zhang L.F., Wang W.J., Cheng J., Shi Y.H., Zhang Q., Dou P., Xu X.H. (2018). Skeleton networks of graphene wrapped double-layered polypyrrole/polyaniline nanotubes for supercapacitor applications. J. Mater. Sci..

[17] Ji T., Tu R., Mu L.W., Lu X.H., Zhu J.H. (2018). Structurally tuning microwave absorption of core/shell structured cnt/polyaniline catalysts for energy efficient saccharide-hmf conversion. Appl. Catal. B Environ..

[18] Sampreeth T., Al-Maghrabi M.A., Bahuleyan B.K., Ramesan M.T. (2018). Synthesis, characterization, thermal properties, conductivity and sensor application study of polyaniline/cerium-doped titanium dioxide nanocomposites. J. Mater. Sci..

[19] Gercek B., Yavuz M., Yilmaz H., Sari B., Unal H.I. (2007). Comparison of electrorheological properties of some polyaniline derivatives. Colloids Surf. A Physicochem. Eng. Asp..

[20] Yilmaz H., Zengin H., Unal H.I. (2012). Synthesis and electrorheological properties of polyaniline/silicon dioxide composite. J. Mater. Sci..

[21] Stenicka M., Pavlinek V., Saha P., Blinova N.V., Stejskal J., Quadrat O. (2008). Conductivity of flowing polyaniline suspensions in electric field. Colloid Polym. Sci..

[22] Cheng Q., Pavlinek V., He Y., Li C., Saha P. (2009). Electrorheological characteristics of polyaniline/titanate composite nanotube suspensions. Colloid Polym. Sci..

[23] Jang W.H., Kim J.W., Choi H.J., Jhon M.S. (2001). Synthesis and electrorheology of camphorsulfonic acid doped polyaniline suspensions. Colloid Polym. Sci..

[24] Kim M.J., Liu Y.D., Choi H.J. (2014). Urchin-like polyaniline microspheres fabricated from self-assembly of polyaniline nanowires and their electro-responsive characteristics. Chem. Eng. J..

[25] Gow C.J., Zukoski C.F. (1990). The electrorheological properties of polyaniline suspensions. J. Colloid Interface Sci..

[26] Jun C.S., Sim B.M., Choi H.J. (2015). Fabrication of electric-stimuli responsive polyaniline/laponite composite and its viscoelastic and dielectric characteristics. Colloids Surf. A Physicochem. Eng. Asp..

[27] Han W.J., Piao S.H., Choi H.J. (2017). Synthesis and electrorheological characteristics of polyaniline@attapulgite nanoparticles via pickering emulsion polymerization. Mater. Lett..

[28] Marins J.A., Giulieri F., Soares B.G., Bossis G. (2013). Hybrid polyaniline-coated sepiolite nanofibers for electrorheological fluid applications. Synth. Met..

[29] Zheng C., Dong Y.Z., Liu Y., Zhao X.P., Yin J.B. (2017). Enhanced stimuli-responsive electrorheological property of poly(ionic liquid)s-capsulated polyaniline particles. Polymers.

[30] Lim G.H., Choi H.J. (2017). Fabrication of self-assembled polyaniline tubes and their electrorheological characteristics. Colloids Surf. A Physicochem. Eng. Asp..

[31] Fang Y., Tan J., Lan T., Foo S.G.F., Pyun D.G., Lim S., Kim D.H. (2018). Universal one-pot, one-step synthesis of core–shell nanocomposites with self-assembled tannic acid shell and their antibacterial and catalytic activities. J. Appl. Polym. Sci..

[32] Zhou T., Zhang T., Zeng Y., Zhang R., Lou Z., Deng J., Wang L. (2018). Structure-driven efficient NiFe_2_O_4_ materials for ultra-fast response electronic sensing platform. Sens. Actuators B Chem..

[33] Liu Y., Hou W.J., Sun H., Cui C., Zhang L.Q., Jiang Y., Wu Y.X., Wang Y.Y., Li J., Sumerlin B.S. (2017). Thiol-ene click chemistry: A biocompatible way for orthogonal bioconjugation of colloidal nanoparticles. Chem. Sci..

[34] Su Y., Guo H., Wang Z., Long Y., Li W., Tu Y. (2018). Au@Cu_2_O core-shell structure for high sensitive non-enzymatic glucose sensor. Sens. Actuators B Chem..

[35] Zhao H., Zhang T., Qi R., Dai J., Liu S., Fei T., Lu G. (2018). Development of solution processible organic-inorganic hybrid materials with core-shell framework for humidity monitoring. Sens. Actuators B Chem..

[36] Zhen W., Ning X., Yang B., Wu Y., Li Z., Lu G. (2018). The enhancement of cds photocatalytic activity for water splitting via anti-photocorrosion by coating Ni_2_P shell and removing nascent formed oxygen with artificial gill. Appl. Catal. B Environ..

[37] Meng C., Zhikun W., Qiang L., Chunling L., Shuangqing S., Songqing H. (2018). Preparation of amino-functionalized Fe_3_O_4_@mSiO_2_ core-shell magnetic nanoparticles and their application for aqueous Fe^3+^ removal. J. Hazard. Mater..

[38] Hussain I., Li Y., Qi J., Li J., Sun X., Shen J., Han W., Wang L. (2018). Synthesis of magnetic yolk-shell mesoporous carbon architecture for the effective adsorption of sulfamethazine drug. Microporous Microporous Mater..

[39] Dai Y.Q., Sun H., Pal S., Zhang Y.L., Park S.W., Kabb C.P., Wei W.D., Sumerlin B.S. (2017). Nera-IR-induced dissociation of thermally-sensitive star polymers. Chem. Sci..

[40] Park S.Y., Cho M.S., Kim C.A., Choi H.J., Jhon M.S. (2003). Polyaniline microsphere encapsulated by poly(methyl methacrylate) and investigation of its electrorheological properties. Colloid Polym. Sci..

[41] Liu Y.D., Kim J.E., Choi H.J. (2011). Core-shell structured monodisperse poly(3,4-ethylenedioxythiophene)/poly(styrenesulfonic acid) coated polystyrene microspheres and their electrorheological response. Macromol. Rapid Commun..

[42] Kim M.W., Moon I.J., Choi H.J., Seo Y. (2016). Facile fabrication of core/shell structured SiO_2_/polypyrrole nanoparticles with surface modification and their electrorheology. RSC Adv..

[43] Kim M.H., Choi H.J. (2017). Core–shell structured semiconducting poly(diphenylamine)-coated polystyrene microspheres and their electrorheology. Polymer.

[44] Park D.E., Chae H.S., Choi H.J., Maity A. (2015). Magnetite-polypyrrole core-shell structured microspheres and their dual stimuli-response under electric and magnetic fields. J. Mater. Chem. C.

[45] Winslow W.M. (1949). Induced fibration of suspensions. J. App. Phys..

[46] Klass D.L., Martinek T.W. (1967). Electroviscous fluids. I. Rheological properties. J. Appl. Phys..

[47] Stangroom J.E. (1983). Electrorheological fluids. Phys. Technol..

[48] Kim Y.D., Klingenberg D.J. (1996). Two roles of nonionic surfactants on the electrorheological response. J. Colloid Interface Sci..

[49] Stenicka M., Pavlinek V., Saha P., Blinova N.V., Stejskal J., Quadrat O. (2009). The electrorheological efficiency of polyaniline particles with various conductivities suspended in silicone oil. Colloid Polym. Sci..

[50] Parthasarathy M., Klingenberg D.J. (1996). Electrorheology: Mechanisms and models. Mater. Sci. Eng. R Rep..

[51] Hao T., Kawai A., Ikazaki F. (1998). Mechanism of the electrorheological effect:  Evidence from the conductive, dielectric, and surface characteristics of water-free electrorheological fluids. Langmuir.

[52] Hao T., Kawai A., Ikazaki F. (1999). Dielectric criteria for the electrorheological effect. Langmuir.

[53] Sedlacik M., Mrlik M., Pavlinek V., Saha P., Quadrat O. (2012). Electrorheological properties of suspensions of hollow globular titanium oxide/polypyrrole particles. Colloid Polym. Sci..

[54] Atten P., Foulc J.N., Felici N. (1994). A conduction model of the electrorheological effect. Int. J. Mod. Phys. B.

[55] Foulc J.N., Atten P., Félici N. (1994). Macroscopic model of interaction between particles in an electrorheological fluid. J. Electrost..

[56] Khusid B., Acrivos A. (1995). Effects of conductivity in electric-field-induced aggregation in electrorheological fluids. Phys. Rev. E.

[57] Cho M.S., Cho Y.H., Choi H.J., Jhon M.S. (2003). Synthesis and electrorheological characteristics of polyaniline-coated poly(methyl methacrylate) microsphere:  Size effect. Langmuir.

[58] Tan Z.J., Zou X.W., Zhang W.B., Jin Z.Z. (1999). Influences of the size and dielectric properties of particles on electrorheological response. Phys. Rev. E.

[59] Wen W., Huang X., Sheng P. (2004). Particle size scaling of the giant electrorheological effect. Appl. Phys. Lett..

[60] Sillars R.W. (1937). The properties of a dielectric containing semiconducting particles of various shapes. Proc. Wirel. Sect. Inst. Electr. Eng..

[61] Wang B., Zhao X. (2005). Core/shell nanocomposite based on the local polarization and its electrorheological behavior. Langmuir.

[62] Liu Y.D., Fang F.F., Choi H.J. (2010). Core–shell structured semiconducting pmma/polyaniline snowman-like anisotropic microparticles and their electrorheology. Langmuir.

[63] Kanu R.C., Shaw M.T. (1998). Enhanced electrorheological fluids using anisotropic particles. J. Rheol..

[64] Wen W.J., Huang X.X., Yang S.H., Lu K.Q., Sheng P. (2003). The giant electrorheological effect in suspensions of nanoparticles. Nat. Mater..

[65] Yin J.B., Zhao X.P. (2004). Giant electrorheological activity of high surface area mesoporous cerium-doped TiO_2_ templated by block copolymer. Chem. Phys. Lett..

[66] Lu K.Q., Shen R., Wang X.Z., Sun G., Wen W.J., Liu J.X. (2007). Polar molecule type electrorheological fluids. Int. J. Mod. Phys. B.

[67] Hong Y.Y., Wen W.J. (2015). Influence of carrier liquid on nanoparticle-based giant electrorheological fluid. J. Intell. Mater. Syst. Struct..

[68] Mai Z.X., Chen J.L., Hu Y., Liu F., Fu B., Zhang H.W., Dong X.M., Huang W.H., Zhou W.Y. (2017). Novel functional mesoporous silica nanoparticles loaded with vitamin e acetate as smart platforms for ph responsive delivery with high bioactivity. J. Colloid Interface Sci..

[69] Yang Y., Lin Y.Z., Di D.H., Zhang X., Wang D., Zhao Q.F., Wang S.L. (2017). Gold nanoparticle-gated mesoporous silica as redox-triggered drug delivery for chemo-photothermal synergistic therapy. J. Colloid Interface Sci..

[70] Muñoz-Pina S., Ros-Lis J.V., Argüelles Á., Coll C., Martínez-Máñez R., Andrés A. (2018). Full inhibition of enzymatic browning in the presence of thiol-functionalised silica nanomaterial. Food Chem..

[71] Feng J.N., She X.J., He X.Y., Zhu J.L., Li Y., Deng C.H. (2018). Synthesis of magnetic graphene/mesoporous silica composites with boronic acid-functionalized pore-walls for selective and efficient residue analysis of aminoglycosides in milk. Food Chem..

[72] Sarkar P., Moyez S.A., Dey A., Roy S., Das S.K. (2017). Experimental investigation of photocatalytic and photovoltaic activity of titania/rice husk crystalline nano-silica hybrid composite. Sol. Energy Mater. Sol. Cells.

[73] Gan C.F., Wang B.F., Huang J.Y., Qileng A., He Z., Lei H.T., Liu W.P., Liu Y.J. (2017). Multiple amplified enzyme-free electrochemical immunosensor based on G-quadruplex/hemin functionalized mesoporous silica with redox-active intercalators for microcystin-LR detection. Biosens. Bioelectron..

[74] Spyrogianni A., Herrmann I.K., Keevend K., Pratsinis S.E., Wegner K. (2017). The silanol content and in vitro cytolytic activity of flame-made silica. J. Colloid Interface Sci..

[75] Ji J.Q., Zeng C.L., Ke Y.C., Pei Y. (2017). Preparation of poly(acrylamide-*co*-acrylic acid)/silica nanocomposite microspheres and their performance as a plugging material for deep profile control. J. Appl. Polym. Sci..

[76] Lengalova A., Pavlinek V., Saha P., Stejskal J., Kitano T., Quadrat O. (2003). The effect of dielectric properties on the electrorheology of suspensions of silica particles coated with polyaniline. Physica A.

[77] Liu B.T., Syu J.R., Wang D.H. (2013). Conductive polyurethane composites containing polyaniline-coated nano-silica. J. Colloid Interface Sci..

[78] Kim M.K., Cho S.H., Song J.Y., Son S., Jang J.S. (2012). Controllable synthesis of highly conductive polyaniline coated silica nanoparticles using self-stabilized dispersion polymerization. ACS Appl. Mater. Interface.

[79] Hong J.Y., Kwon E.B., Jang J.S. (2009). Fabrication of silica/polythiophene core/shell nanospheres and their electrorheological fluid application. Soft Matter.

[80] Zhou L., Tan G.X., Ouyang K., Liu Y., Ning C.Y. (2015). Highly water-dispersible, highly conductive, and biocompatible polypyrrole-coated silica particles stabilized and doped by chondroitin sulfate. Part. Part. Syst. Charact..

[81] Kwon S.H., Liu Y.D., Choi H.J. (2015). Monodisperse poly(2-methylaniline) coated polystyrene core–shell microspheres fabricated by controlled releasing process and their electrorheological stimuli-response under electric fields. J. Colloid Interface Sci..

[82] Sharp Norton J.C., Han M.G., Creager S., Foulger S.H. (2012). Electrochemical redox of pedot-coated core-shell silica spheres stabilized in a peg-based hydrogel maxtrix: Modulation of the optical properties by doping with various oxidative mediators. Int. J. Electrochem. Sci..

[83] Trlica J., Saha P., Quadrat O., Stejskal J. (2000). Electrorheology of polyaniline-coated silica particles in silicone oil. J. Phys. D Appl. Phys..

[84] Jang J.S., Ha J.S., Lim B.K. (2006). Synthesis and characterization of monodisperse silica-polyaniline core-shell nanoparticles. Chem. Commun..

[85] Hong J.Y., Jang J.S. (2010). A comparative study on electrorheological properties of various silica-conducting polymer core-shell nanospheres. Soft Matter.

[86] Park D.E., Choi H.J., Vu C.M. (2016). Stimuli-responsive polyaniline coated silica microspheres and their electrorheology. Smart Mater. Struct..

[87] Uma K., Arjun N., Pan G.T., Yang T.C.K. (2017). The photodeposition of surface plasmon ag metal on SiO_2_@α-Fe_2_O_3_ nanocomposites sphere for enhancement of the photo-fenton behavior. Appl. Surf. Sci..

[88] Silvestri S., Foletto E.L. (2017). Preparation and characterization of Fe_2_O_3_/TiO_2_/clay plates and their use as photocatalysts. Ceram. Int..

[89] Predescu A., Nicolae A. (2012). Adsorption of Zn, Cu and Cd from waste waters by means of maghemite nanoparticles. UPB Sci. Bull. Ser. B.

[90] Berry C.C., Curtis A.S.G. (2003). Functionalisation of magnetic nanoparticles for applications in biomedicine. J. Phys. D Appl. Phys..

[91] Zhong B., Wang C.J., Yu Y.L., Xia L., Wen G.W. (2017). Facile fabrication of carbon microspheres decorated with B(OH)_3_ and α-Fe_2_O_3_ nanoparticles: Superior microwave absorption. J. Colloid Interface Sci..

[92] Liu C., Wang Y.L., Zhao P.L., Li W.B., Wang Q.J., Sun P., Chuai X.H., Lu G.Y. (2017). Porous α-Fe_2_O_3_ microflowers: Synthesis, structure, and enhanced acetone sensing performances. J. Colloid Interface Sci..

[93] Zhu J.B., Wei L.P., Hu J., Xue C.T. (2017). Anchoring iron oxide nanoparticles on polypyrrole/rGO derived nitrogen-doped carbon as lithium-ion battery anode. J. Alloys Compd..

[94] Roy M., Naskar M.K. (2016). Alkali metal ion induced cube shaped mesoporous hematite particles for improved magnetic properties and efficient degradation of water pollutants. Phys. Chem. Chem. Phys..

[95] Wang P., Zheng Z.K., Cheng X.L., Sui L.L., Gao S., Zhang X.F., Xu Y.M., Zhao H., Huo L.H. (2017). Ionic liquid-assisted synthesis of [small alpha]-Fe_2_O_3_ mesoporous nanorod arrays and their excellent trimethylamine gas-sensing properties for monitoring fish freshness. J. Mater. Chem. A.

[96] Lin Q., Chen Y.B., Zhong Y.J., Li L., Zhou W., Shao Z.P. (2017). Pine-leaf-shaped α-Fe_2_O_3_micro/nanostructures with a preferred orientation along the (110) plane for efficient reversible lithium storage. ChemElectroChem.

[97] Wang B.H., Sun J.Q., Abbas M., Liu Y.T., Kong F.H., Xiao H.C., Chen J.G. (2017). A novel hydrothermal approach for the synthesis of flower-like Fe_2_O_3_/Fe foam nanocrystals and their superior performance in fisher–tropsch synthesis. Catal. Lett..

[98] Tian X.L., He K., Wang B.X., Yu S.S., Hao C.C., Chen K.Z., Lei Q.Q. (2016). Flower-like Fe_2_O_3_/polyaniline core/shell nanocomposite and its electroheological properties. Colloids Surf. A Physicochem. Eng. Asp..

[99] Du J., Ding Y., Guo L.G., Wang L., Fu Z.B., Qin C.Q., Wang F., Tao X.Y. (2017). Micro-tube biotemplate synthesis of Fe_3_O_4_/C composite as anode material for lithium-ion batteries. Appl. Surf. Sci..

[100] Bera R., Das A.K., Maitra A., Paria S., Karan S.K., Khatua B.B. (2017). Salt leached viable porous Fe_3_O_4_ decorated polyaniline—SWCNH/PVDF composite spectacles as an admirable electromagnetic shielding efficiency in extended Ku-band region. Compos. B Eng..

[101] Mashkani M., Mehdinia A., Jabbari A., Bide Y., Nabid M.R. (2018). Preconcentration and extraction of lead ions in vegetable and water samples by N-doped carbon quantum dot conjugated with Fe_3_O_4_ as a green and facial adsorbent. Food Chem..

[102] Jung K.W., Choi B.H., Ahn K.H., Lee S.H. (2017). Synthesis of a novel magnetic Fe_3_O_4_/γ-Al_2_O_3_ hybrid composite using electrode-alternation technique for the removal of an azo dye. Appl. Surf. Sci..

[103] Zhu S.M., Dong B.Z., Yu Y.H., Bu L.J., Deng J., Zhou S.Q. (2017). Heterogeneous catalysis of ozone using ordered mesoporous Fe_3_O_4_ for degradation of atrazine. Chem. Eng. J..

[104] Jiang X.Y., Li L.L., Cui Y.R., Cui F.L. (2017). New branch on old tree: Green-synthesized rGO/Fe_3_O_4_ composite as a photo-Fenton catalyst for rapid decomposition of methylene blue. Ceram. Int..

[105] Gao Y.L., Zhu G.M., Xu S.G., Ma T.T., Nie J. (2018). Biodegradable magnetic-sensitive shape memory poly(ɛ-caprolactone)/Fe_3_O_4_ nanocomposites. J. Appl. Polym. Sci..

[106] Wu J.M., Ye Z.M., Liu W.X., Liu Z.F., Chen J. (2017). The effect of GO loading on electromagnetic wave absorption properties of Fe_3_O_4_/reduced graphene oxide hybrids. Ceram. Int..

[107] Long J., Zhang B., Li X.F., Zhan X.B., Xu X.M., Xie Z.J., Jin Z.Y. (2018). Effective production of resistant starch using pullulanase immobilized onto magnetic chitosan/Fe_3_O_4_ nanoparticles. Food Chem..

[108] Shah M.T., Alveroglu E. (2017). Synthesis and characterization of magnetite nanoparticles having different cover layer and investigation of cover layer effect on the adsorption of lysozyme and bovine serum albumin. Mater. Sci. Eng. C.

[109] Sim B., Chae H.S., Choi H.J. (2015). Fabrication of polyaniline coated iron oxide hybrid particles and their dual stimuli-response under electric and magnetic fields. Express Polym. Lett..

[110] Yu X.Q., Lin D.M., Li P., Su Z.Q. (2017). Recent advances in the synthesis and energy applications of TiO_2_-graphene nanohybrids. Sol. Energy Mater. Sol. Cells.

[111] Wan T.T., Ramakrishna S., Liu Y. (2018). Recent progress in electrospinning TiO_2_ nanostructured photo-anode of dye-sensitized solar cells. J. Appl. Polym. Sci..

[112] Sornalingam K., McDonagh A., Zhou J.L., Johir M.A.H., Ahmed M.B. (2018). Photocatalysis of estrone in water and wastewater: Comparison between Au-TiO_2_ nanocomposite and TiO_2_, and degradation by-products. Sci. Total Environ..

[113] Sakkas V.A., Sarro M., Kalaboka M., Santoro V., Albanis T., Calza P., Medana C. (2017). Evaluating the photocatalytic treatment of stevioside by TiO_2_ in different aqueous matrices and identification of transformation products. Sci. Total Environ..

[114] Yu L.F., Zhang S.M., Zhang M., Chen J.D. (2017). Superhydrophobicity construction with dye-sensitised TiO_2_ on fabric surface for both oil/water separation and water bulk contaminants purification. Appl. Surf. Sci..

[115] Li Z.Q., Qi M.Y., Tu C.Y., Wang W.P., Chen J.R., Wang A.J. (2017). Highly efficient removal of chlorotetracycline from aqueous solution using graphene oxide/TiO_2_ composite: Properties and mechanism. Appl. Surf. Sci..

[116] Wang B.X., Liu C.J., Yin Y.C., Yu S.S., Chen K.Z., Liu P.B., Liang B. (2013). Double template assisting synthesized core–shell structured titania/polyaniline nanocomposite and its smart electrorheological response. Compos. Sci. Technol..

[117] Tian X.L., He K., Wang C.W., Wen Q.K., Wang B.X., Yu S.S., Hao C.C., Chen K.Z., Lei Q.Q. (2016). Preparation and electrorheological behavior of anisotropic titanium oxide/polyaniline core/shell nanocomposite. Compos. Sci.Technol..

[118] DeSimone J.M., Maury E.E., Menceloglu Y.Z., McClain J.B., Romack T.J., Combes J.R. (1994). Dispersion polymerizations in supercritical carbon dioxide. Science.

[119] Shen S., Sudol E.D., El-Aasser M.S. (1994). Dispersion polymerization of methyl methacrylate: Mechanism of particle formation. J. Polym. Sci. A Polym. Chem..

[120] Cho Y.H., Cho M.S., Choi H.J., Jon M.S. (2002). Electrorheological characterization of polyaniline-coated poly(methyl methacrylate) suspensions. Colloid Polym. Sci..

[121] Lee I.S., Cho M.S., Choi H.J. (2005). Preparation of polyaniline coated poly(methyl methacrylate) microsphere by graft polymerization and its electrorheology. Polymer.

[122] Fang F.F., Liu Y.D., Lee I.S., Choi H.J. (2011). Well controlled core/shell type polymeric microspheres coated with conducting polyaniline: Fabrication and electrorheology. RSC Adv..

[123] Zhang W.L., Piao S.H., Choi H.J. (2013). Facile and fast synthesis of polyaniline-coated poly(glycidyl methacrylate) core-shell microspheres and their electro-responsive characteristics. J. Colloid Interface Sci..

[124] Barthet C., Armes S.P., Lascelles S.F., Luk S.Y., Stanley H.M.E. (1998). Synthesis and characterization of micrometer-sized, polyaniline-coated polystyrene latexes. Langmuir.

[125] Kim Y.H., Park B.J., Choi H.J., Choi S.B. (2009). Preparation of polystyrene/polyaniline composite particles and their electrorheology. J. Phys. Conf. Ser..

[126] Liu Y.D., Park B.J., Kim Y.H., Choi H.J. (2011). Smart monodisperse polystyrene/polyaniline core-shell structured hybrid microspheres fabricated by a controlled releasing technique and their electro-responsive characteristics. J. Mater. Chem..

[127] Piao S.H., Gao C.Y., Choi H.J. (2017). Sulfonated polystyrene nanoparticles coated with conducting polyaniline and their electro-responsive suspension characteristics under electric fields. Polymer.

[128] Kim D.D., Tian Y., Choi H.J. (2015). Seeded swelling polymerized sea urchin-like core-shell typed polystyrene/polyaniline particles and their electric stimuli-response. RSC Adv..

[129] Gandla  D., Putta C., Ghosh  S., Hazra B.K. (2016). Carbon sphere-polyaniline composite: A fluorescent scaffold for proliferation of adipose derived stem cells and its cellular uptake. Chem. Sel..

[130] Wei Y., Wang J.G., Jia X.R., Yeh J.M., Spellane P. (1995). Polyaniline as corrosion protection coatings on cold rolled steel. Polymer.

[131] Helal A., Qian B., McKinley G.H., Hosoi A.E. (2016). Yield hardening of electrorheological fluids in channel flow. Phys. Rev. Appl..

[132] Engelmann B., Hiptmair R., Hoppe R.H.W., Mazurkevitch G. (2000). Numerical simulation of electrorheological fluids based on an extended bingham model. Comput. Vis. Sci..

[133] Melek Y., Heming D. (1999). Magnetorheological and electrorheological materials in adaptive structures and their performance comparison. Smart Mater. Struct..

[134] Cho M.S., Choi H.J., Jhon M.S. (2005). Shear stress analysis of a semiconducting polymer based electrorheological fluid system. Polymer.

[135] Zhang K., Kim S.Y., Jariyasakoolroj P., Chirachanchai S., Choi H.J. (2017). Stimuli-response of chlorosilane-functionalized starch suspension under applied electric fields. Polym. Bull..

[136] Bae D.H., Choi H.J., Choi K.S., Nam J.D., Islam M.S., Kao N. (2017). Fabrication of phosphate microcrystalline rice husk based cellulose particles and their electrorheological response. Carbohydr. Polym..

[137] An J.S., Moon I.J., Kwon S.H., Choi H.J. (2017). Swelling-diffusion-interfacial polymerized core-shell typed polystyrene/poly(3,4-ethylenedioxythiophene) microspheres and their electro-responsive characteristics. Polymer.

[138] Choi H.J., Lee Y.H., Kim C.A., Jhon M.S. (2000). Microencapsulated polyaniline particles for electrorheological materials. J. Mater. Sci. Lett..

[139] Sim B.M., Zhang W.L., Choi H.J. (2015). Graphene oxide/poly(2-methylaniline) composite particle suspension and its electro-response. Mater. Chem. Phys..

[140] Choi H.J., Cho M.S., Kim J.W., Kim C.A., Jhon M.S. (2001). A yield stress scaling function for electrorheological fluids. Appl. Phys. Lett..

[141] Zhang W.L., Jiang D.G., Wang X.X., Hao B.N., Liu Y.D., Liu J.Q. (2017). Growth of polyaniline nanoneedles on MoS_2_ nanosheets, tunable electroresponse, and electromagnetic wave attenuation analysis. J. Phys. Chem. C.

[142] Zhang W.L., Tian Y., Liu Y.D., Song Z.Q., Liu J.Q., Choi H.J. (2016). Large scale and facile sonochemical synthesis of magnetic graphene oxide nanocomposites and their dual electro/magneto-stimuli responses. RSC Adv..

[143] Cole K.S., Cole R.H. (1941). Dispersion and absorption in dielectrics I. Alternating current characteristics. J. Chem. Phys..

[144] Kim M.H., Bae D.H., Choi H.J., Seo Y.S. (2017). Synthesis of semiconducting poly(diphenylamine) particles and analysis of their electrorheological properties. Polymer.

